# Carbon nitride supported copper nanoparticles: light-induced electronic effect of the support for triazole synthesis

**DOI:** 10.1098/rsos.160580

**Published:** 2016-11-16

**Authors:** Debkumar Nandi, Abu Taher, Rafique Ul Islam, Samarjeet Siwal, Meenakshi Choudhary, Kaushik Mallick

**Affiliations:** Department of Chemistry, University of Johannesburg, PO Box 524, Auckland Park 2006, South Africa

**Keywords:** carbon nitride, copper nanoparticle, UV-induced electronic effect, triazole synthesis

## Abstract

The composite framework of graphitic carbon nitride (*g*CN) supported copper nanoparticle can act as a high-performance photoreactor for the synthesis of 1,2,3-triazole derivatives under light irradiation in the absence of alkaline condition. The photoactivity of *g*CN originates from an electron transition from the valence band to the conduction band, in the presence of photon energy, and the hot electron acts as a scavenger of the terminal proton of the alkyne molecule to facilitate the formation of copper acetanilide complex. In this study, we have performed the experiment under a different photonic environment, including dark condition, and in the presence and absence of base. A comparative study was also executed using Cu-TiO_2_ system, as a reference material, in the support of our proposed mechanism. The recycling performance and the photocorrosion effect of the catalyst have also been reported in this study.

## 1. Introduction

The copper-catalysed regioselective 1,3-dipolar cycloaddition of azide and alkyne, to produce five-membered nitrogen heterocyclic 1,2,3-triazoles compounds, is commonly known as the Click reaction [1,2], which has tremendous potential in biology and medicinal applications [3]. The Click products also have industrial applications such as corrosion inhibitors [4], pharmaceuticals, agrochemicals [5] and luminescent materials [6].

The Click reaction can be performed using wide varieties of copper species. Early studies on developing heterogeneous catalysts for the Click reaction based on mixed Cu/Cu-oxide nanoparticles, which yield regioselective product [7], stable poly(*N*-vinyl-2-pyrrolidone) protected copper nanoparticles for highly recyclable purpose [8], graphene oxide supported copper oxide composite for green catalysis process [9], catalyst, Al_2_O_3_-Cu, synthesized through ball-milling process for a solvent-free condition [10], recoverable SiO_2_-Cu composite catalyst for microwave irradiation technique [11], ligand-free zeolite based copper (I) catalyst for the single regioisomeric product [12], copper nanoparticles for short reaction duration with excellent yields [13] and one-pot reaction using Cu(II)-hydrotalcite catalysed eco-friendly route [14], have been reported. Protocols have also been documented for the photo-induced formation of Cu(I), active form of the catalyst, using graphitic carbon nitride (*g*CN) [15], fullerene [16] and photosensitizer ruthenium [17] as a photocatalyst, from the Cu(II) precursor in the presence of amine as a base molecule for azide–alkyne cycloaddition reaction. Copper-based nano-catalysts have also been reported in the literature for various kinds of organic transformation reactions[18,19].

Semiconductor materials are attractive to scientists for their electronic and optoelectronic application, through the band-gap engineering in the presence of photon energy, using high-energy conduction band (CB) electrons and photo-generated holes. *g*CN, which is composed of carbon and nitrogen with a unique framework of tri-s-triazine linked by tertiary amines, has received much attention as a medium band-gap photoactive material with promising photocatalytic properties [20–23]. Besides that, carbon nitride has a high thermal and chemical stability and amenability towards chemical modification, which makes it suitable for a range of applications. *g*CN with platinum nanoparticle composite catalyst exhibits activities for solar-driven hydrogen production [24]. *g*CN-based materials are also evidenced for the oxidation of alkanes [25], olefins [23] and alcohols [26]. Under the illumination of visible light, mesoporous-C_3_N_4_ can also promote the oxidation of amines [27]. Mesoporous carbon nitride supported Pd-nanoparticles, catalysed by Suzuki coupling reaction, have been reported with excellent yield at room temperature under light irradiation through the photo-induced charge transfer from *g*CN to palladium [28].

Motivated by the above reports, we have carried out the experiment to explore the photonic effect of the support material for *g*CN-supported copper nanoparticles on the azide–alkyne cycloaddition reactions [1,2] for the synthesis of 1,4-disubstituted 1,2,3-triazole compounds. In connection with our ongoing research on the development of effective catalysts for organic transformation reactions[29–32], we have reported *in situ* catalyst formation and cycloaddition reaction in a one-pot method [33] and Cu(I)-polyaminobenzoic acid catalysed azide–alkyne cycloaddition reaction [34]. Again, a solvent-less microwave irradiation technique has also been reported for a 1,3-dipolar cycloaddition reaction between terminal alkynes and azides to synthesize 1,2,3-triazoles using a polymer-supported copper (I) composite, fabricated using a one-step chemical synthesis route under ambient conditions [35].

In this report, graphitic carbon nitride supported copper nanoparticles (Cu-*g*CN) have been synthesized using the single-step borohydride reduction technique [36]. The synthesized composite material (Cu-*g*CN) has been used as a catalyst for triazole synthesis under different illumination conditions, ultraviolet (UV), daylight (DL) and dark (D), in the presence and absence of a base (triethylamine, Et_3_N). Additionally, we compare the photocatalyst performance of the Cu-*g*CN with another photoactive material (Cu-TiO_2_) for the same reaction to find the role of amine group in the *g*CN structure for triazole formation under dark conditions in the presence and absence of a base.

## 2. Experimental details

### 2.1. Materials

All the chemicals and the solvents used for this experiment were of analytical purity and used without further purification. Ultra-pure water (specific resistivity > 17 MΩ cm) was used in this experiment wherever required.

### 2.2. Synthesis

In this work, *g*CN was synthesized using urea as the precursor at 550°C temperature in a muffle furnace and then Cu-*g*CN (5.0 mol% of Cu loading) was prepared using copper nitrate precursor following the single-step borohydride reduction technique at room temperature. In a similar way, Cu-TiO_2_ (5.0 mol% of Cu loading) was also synthesized using the precursors of copper nitrate and TiO_2_ powder (Degussa, P-25).

### 2.3. General procedure for the triazole synthesis

In a 25 ml cylindrical quartz cell or round bottom flask, azide **1** (1 equiv.) and alkyne **2** (1 equiv.) were charged in 4 ml methanol in the presence or absence of Et_3_N (1 equiv.) in different photonic environments, such as ultraviolet, daylight and dark. To this reaction mixture, 3 mg of Cu-*g*CN catalyst was added and stirred at room temperature. The reaction mixture was stirred for 1 h and progress of the reaction was monitored using thin layer chromatography (TLC) technique. After completion, the reaction mixture was filtered and dried under residue pressure. The dried gummy mass was diluted with 20 ml of distilled water; the solution was extracted with ethyl acetate (3 × 20 ml). The organic layer was separated and dried over anhydrous MgSO_4_. Combined organic layer was concentrated in vacuum to give the corresponding triazoles, which were further purified by recrystallization or by column chromatography technique.

### 2.4. Luminescence condition

Philips UV-C (germicidal) lamp was used as the source of UV light and an optical power meter (Newport) showed the light intensity value was 40 mW cm^−2^ adjacent to the quartz reaction chamber. The optical intensity was also measured during the daylight experiment and was found to be 3.5 mW cm^−2^ inside the fume hood.

### 2.5. Material characterization

Transmission electron microscopy (TEM) studies were performed at an acceleration voltage of 197 kV by using a Philips CM200 TEM instrument equipped with a LaB_6_ source. The TEM samples were prepared by depositing small amount of synthesized material onto a TEM grid (200 mesh size Cu-grid) coated with a lacy carbon film. The X-ray diffraction (XRD) patterns were recorded on a Shimadzu XD-3A X-ray diffractometer operating at 20 kV using Cu-Kα radiation (*k* = 0.1542 nm). The measurements were performed over a diffraction angle range of 2*θ* = 20°–80°. X-ray photoelectron spectra (XPS) were collected in an ultra high vacuum (UHV) chamber attached to a Physical Electronics 560 ESCA/SAM instrument. Fourier transform infrared spectroscopy (FTIR) spectra were collected using a Shimadzu IRAffinity-1 with a spectral resolution of 0.5 cm^−1^. The UV-vis spectra were measured using a Shimadzu UV-1800 spectrophotometer using with a quartz cuvette.

## 3. Results

**Figure 1.**
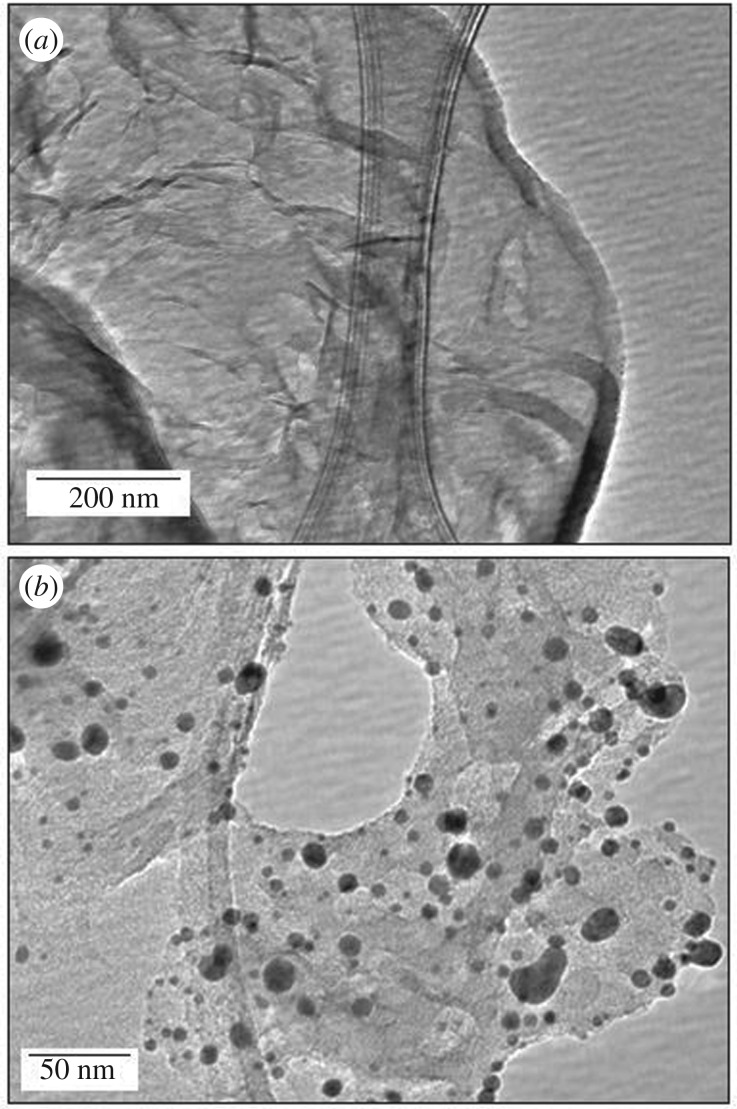
The TEM images of the (*a*) *g*CN and (*b*) Cu-*g*CN. In the Cu-*g*CN sample, a wide size distribution has been noted within the range of 5–20 nm.

**Figure 2:**
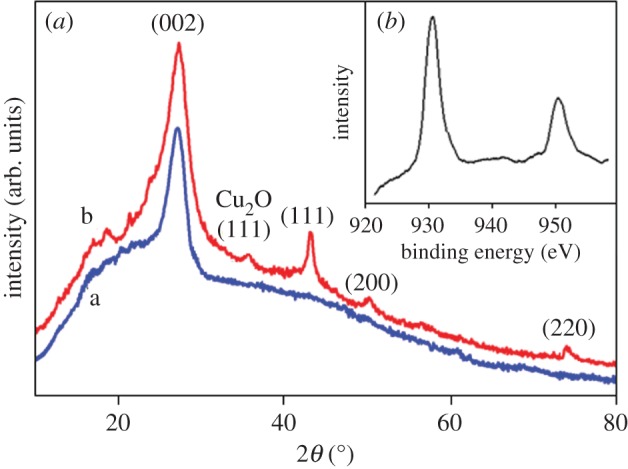
(*a*) The XRD patterns of a, *g*CN, with the peak at 27.33°, indexed as the (002), and b, Cu-*g*CN, where beside carbon nitride peak at 27.33° other three peaks at 43.18°, 50.20° and 74.06° were assigned for the (111), (200) and (220) crystal face of metallic copper. The peak at 35.80°, in b, is for the (111) crystal face of Cu_2_O. (*b*) The X-ray photoelectron spectra of Cu 2p and the peaks at 932.6 and 952.4 eV correspond to the signals of 2p*_3/2_* and 2p*_l/2_*, respectively. The core-level Cu 2p*_3/2_* spectrum at 932.6 eV indicates the metallic nature of the copper nanoparticles in the Cu*-g*CN sample.

The TEM images of the *g*CN support and Cu-*g*CN are shown in figure 1*a*,*b*, respectively. In the copper nanoparticles, a large size distribution, within the range of 5–20 nm, has been noted in figure 1*b*. Some of the agglomerated particles have also been observed in the TEM image of the Cu-*g*CN sample. The phase of the synthesized products was characterized by XRD analysis. In the XRD pattern, figure 2*a* a, the most intense peak at 27.33°, corresponding to an interlayer stacking distance of *d*_002_ = 0.326 nm, and can be indexed as the (002) stacking peak of the conjugated aromatic ring [37]. Figure 2*a* b indicates the XRD pattern of the Cu*-g*CN; besides the carbon nitride peak at 27.33°, the other three peaks at 43.18°, 50.20° and 74.06° can be assigned to the (111), (200) and (220) crystal face of metallic copper (JCPDS number: 04-0836). In the XRD pattern, an additional low-intensity reflections peak has been observed, for the Cu*-g*CN sample, at about 35.80°, which can be assigned to the (111) crystal face for Cu_2_O, indicating surface oxidation of the Cu particles. Unfortunately, it was not possible to perform the XRD analysis under inert atmosphere and the formation of Cu(I) species could be the effect of aerial oxidation of copper nanoparticles. The narrow scan for the XPS of Cu 2p is shown in figure 2*b*. The peaks appearing at 932.6 and 952.4 eV correspond to the signals of 2p*_3/2_* and 2p*_l/2_*, respectively. The symmetric nature of the core-level Cu 2p*_3/2_* spectrum at 932.6 eV indicates the presence of only metallic copper in the Cu*-g*CN sample. The evidence of formation of Cu(I) species, from Cu_2_O as indicated in the XRD pattern, is not visible in the XPS signal probably due to the X-ray-induced reduction of Cu(I) species [38].

**Figure 3.**
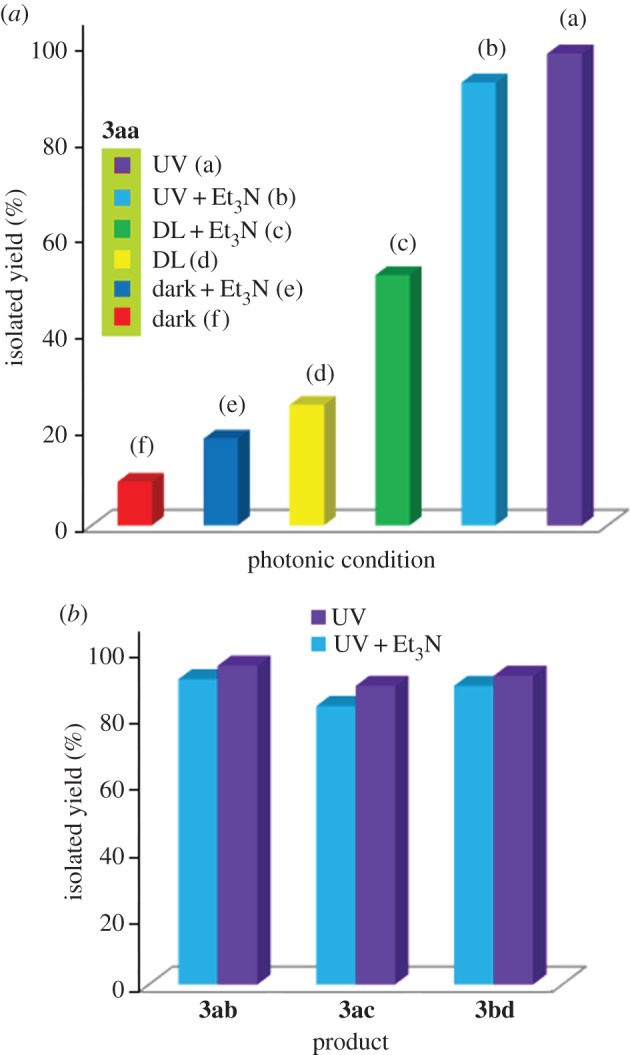
Effect of photon and base on the triazole formation reactions: a graphical representation.

The synthesized Cu-*g*CN sample was used as a catalyst for a model cycloaddition reaction, figure 3, between 1-(azidomethyl)-4-methylbenzene (**1a**) and 1-ethynyl-4-methylbenzene (**2a**) in the presence of daylight. We found that a yield of 25% of desired product, 1-(4-methylbenzyl)-4-*p*-tolyl-1*H*-1,2,3-triazole (**3aa**), has been obtained at 120 min in the presence of methanol as a solvent. On the basis of several experiments, we have recognized that the methanol was the best solvent as compared with the other solvents (CHCl_3_, MeOH, MeOH : H_2_O in different ratio, H_2_O and THF) used for the reaction. In the next step, the effect of base was examined for the same reaction and we found the presence of Et_3_N produced the yield of 52% of the product (**3aa**), in methanol, for the same period of time (120 min) and has the superior performance to other tested bases, such as, K_2_CO_3_, Cs_2_CO_3_, KOH, N_2_H_4_ · H_2_O, di-ethyl amine, di-isopropyl amine. When the same reaction was performed under dark conditions both in the presence and absence of the base (Et_3_N), the triazole product (**3aa**) was obtained with the yield of 18 and 9%, respectively. The most fascinating result was obtained when the above reaction was performed under UV illumination condition where 98% cycloaddition product (**3aa**) was formed in the absence of the base and 92% product was obtained in the presence of base. The above experimental results are shown graphically (figure 3*a*) and in table 1. It is also important to mention that *g*CN alone was not active for the reaction, in other words, in the absence of copper no coupled product has been noted.

**Table 1.** Optimization of photonic effect in triazole synthesis^i,ii^.

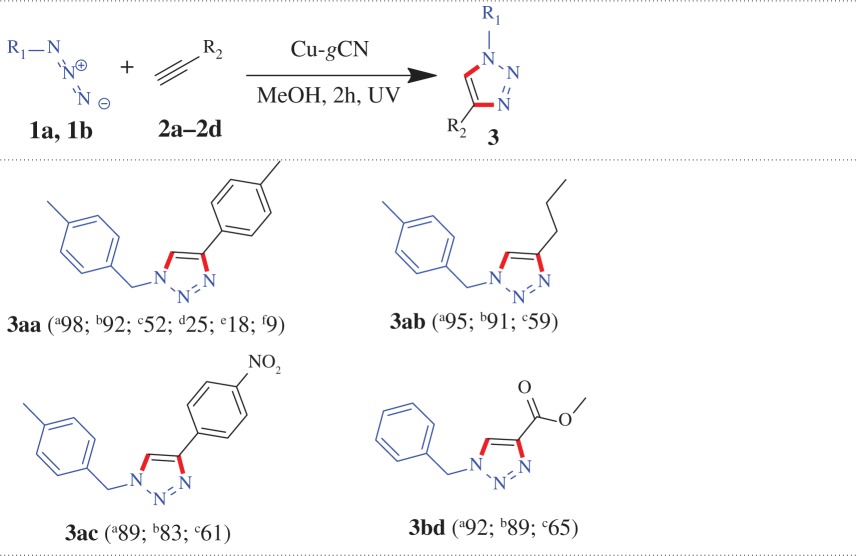

^i^Reaction conditions: azide (1.0 mmol), alkyne (1.0 mmol), MeOH (4.0 ml), Cu-*g*CN (5 mg), in UV light.

^ii^Isolated yields. a = UV, b = UV + Et_3_N (1.0 mmol), c = daylight + Et_3_N (1.0 mmol), d = daylight, e = dark + Et_3_N (1.0 mmol), f = dark.

**Figure 4.**
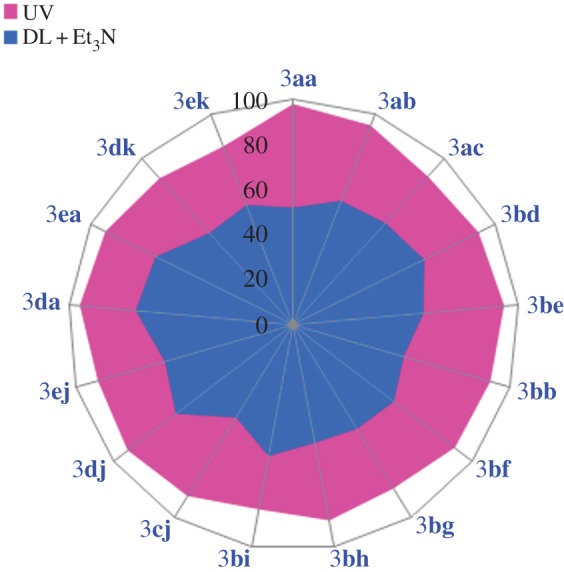
Substrate scope of the reaction under UV and daylight conditions: a yield comparison study.

From the above experiment, we found that under UV illumination condition the base has a negative effect, though marginal, on the amount of the product formation. To confirm that, we have performed three more reactions under the same condition (under UV irradiation, in the presence and absence of base) where a similar trend has also been observed for the products **3ab**, **3ac** and **3bd**, as represented graphically in figure 3*b*, and in table 1 (entries: 2–4). When, 1-(azidomethyl)-4-methylbenzene (**1a**) separately reacted with pent-1-yne (**2b**) and 1-ethynyl-4-nitrobenzene (**2c**) using Cu-*g*CN as a catalyst under UV illumination condition, the products 1-(4-methylbenzyl)-4-propyl-1*H*-1,2,3-triazole (**3ab**) and 1-(4-methylbenzyl)-4-(4-nitrophenyl)-1*H*-1,2,3-triazole (**3ac**) were obtained with the yield of 95 and 89%, in the absence of triethylamine, and 91 and 83%, in the presence of triethylamine, respectively. Again, azidomethyl benzene (**1b**) and methyl propiolate (**2d**) formed the cycloaddition product methyl 1-benzyl-1*H*-1,2,3-triazole-4-carboxylate (**3bd**) under UV illumination condition and produced 92 and 89% yields in the absence and presence of triethylamine, respectively. We also have explored the above reactions under ambient photonic conditions, ‘daylight + base’ (DLB), and found the cycloaddition products **3ab**, **3ac** and **3bd** have been formed with the yield of 59, 61 and 65%, respectively, (the results are summarized graphically (figure 4) and also in table 1).

We also have explored the versatility of the reaction for the other structurally diverse azides and alkynes under two different optical conditions (UV and DLB), where the maximum yields were obtained (figure 4 and table 2). When, (azidomethyl) benzene (**1b**) reacted with alkyne ester such as methyl propiolate (**2d**) and ethyl propiolate (**2e**), the products formed methyl 1-benzyl-1*H*-1,2,3-triazole-4-carboxylate (**3bd**) with the yield of 92 and 65% and ethyl 1-benzyl-1*H*-1,2,3-triazole-4-carboxylate (**3be**) with the yield of 94 and 58%, under UV and DLB conditions, respectively. A similar trend has been observed when azide molecule reacted with aliphatic alkyne and alkyne substituted with both lactone and aliphatic cyclic alcohol systems. During the reaction between (azidomethyl) benzene (**1b**) and aliphatic alkynes (pent-1-yne, **2b**, and hex-1-yne, **2f**) the products 1-benzyl-4-propyl-1*H*-1,2,3-triazole (**3bb**) with the yield of 91 and 51% and 1-benzyl-4-butyl-1*H*-1,2,3-triazole (**3bf**) with the yield of 90 and 56% have been formed under UV and DLB conditions, respectively. The same azide molecule coupled with alkyne substituted lactone (**2 g**) to produce 4-((1-benzyl-1*H*-1,2,3-triazol-4-yl)methoxy)-6-methyl-2H-pyran-2-one (**3bg**) with the yield of 85 and 55% under UV and DLB conditions, respectively. Aliphatic cyclic alcohols, such as, 1-ethynylcyclopentanol (**2h**) and 1-ethynylcyclohexanol (**2i**) when coupled with (azidomethyl) benzene (**1b**) form the cyclo-products of 1-((1-benzyl-1*H*-1,2,3-triazol-4-yl)methyl) cyclopentanol (**3bh**) and 1-(1-benzyl-1*H*-1,2,3-triazol-4-yl) cyclohexanol (**3bi**) with the yield of 88 and 53%, for both the cases, under UV and DLB conditions, respectively. The reaction between 1-(azidomethyl)-2-bromobenzene (**1c**) and phenylacetelene (**2j**) formed the cycloaddition product 1-(2-bromobenzyl)-4-phenyl-1*H*-1,2,3-triazole (**3cj**) with the yield of 89 and 48%, under UV and DLB condition, respectively.

**Table 2.** Substrate scope for Cu-*g*CN catalysed triazole synthesis in different reaction conditions^i,ii^.

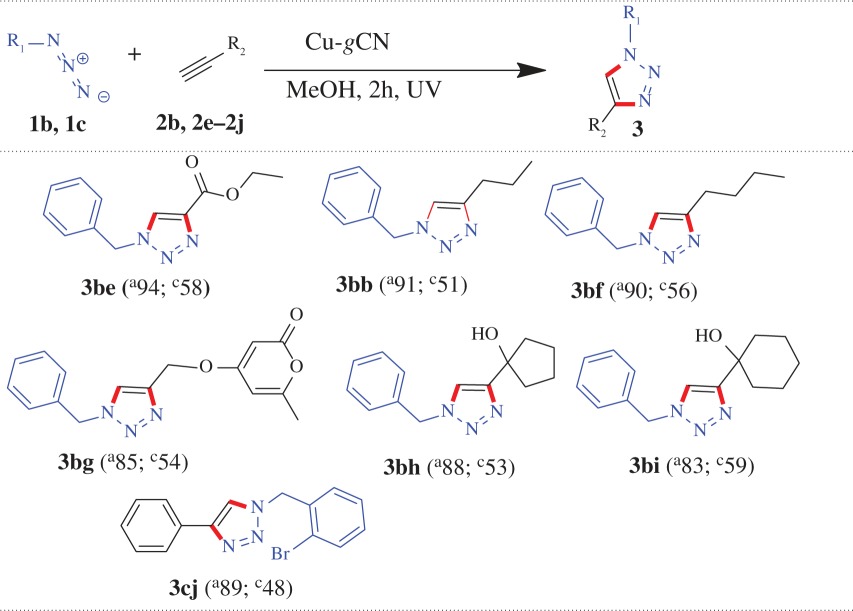

^i^Reaction conditions: azide (1.0 mmol), alkyne (1.0 mmol), MeOH (4.0 ml), Cu-*g*CN (5 mg), in UV light.

^ii^Isolated yields (%), a = UV, c = daylight + Et_3_N (1.0 mmol).

In this study, we have extended our experiments for glucose and galactose substituted azide molecule and we have found that the substituted azide molecule coupled with alkyne molecules to form β-d- glucopyranoside and β-d-galactopyranoside substituted 1,2,3-triazole moieties, respectively, under the UV and DLB conditions in the presence of Cu-*g*CN catalyst. A comparative study shows that the reaction under UV exposure has superior performance than the reaction under daylight in the presence of triethyl amine (DLB) condition. When 1-azido-2,3,4,6-tetra-*O*-acetyl-β-d-glucopyranose (**1d**) reacted with phenylacetylene (**2j**) and its derivatives (1-ethynyl-4-methylbenzene (**2a**) and 1-ethynyl-4-methoxy-2-methylbenzene (**2k**)) the products **3dj**, **3da** and **3dk** are formed with the yield of 92, 95 and 88% under UV conditions and 65, 70 and 55% under DLB conditions, respectively (table 3 and figure 4). A similar trend has also been found when 1-azido-2,3,4,6-tetra-*O*-acetyl-β-d-galactopyranose (**1e**) reacted with alkyne molecules (**2j**, **2a** and **2k**) (table 3 and figure 4).

**Table 3.** Substrate scope for Cu-*g*CN catalysed triazole synthesis of sugar azides with alkynes in different reaction conditions^i,ii^.

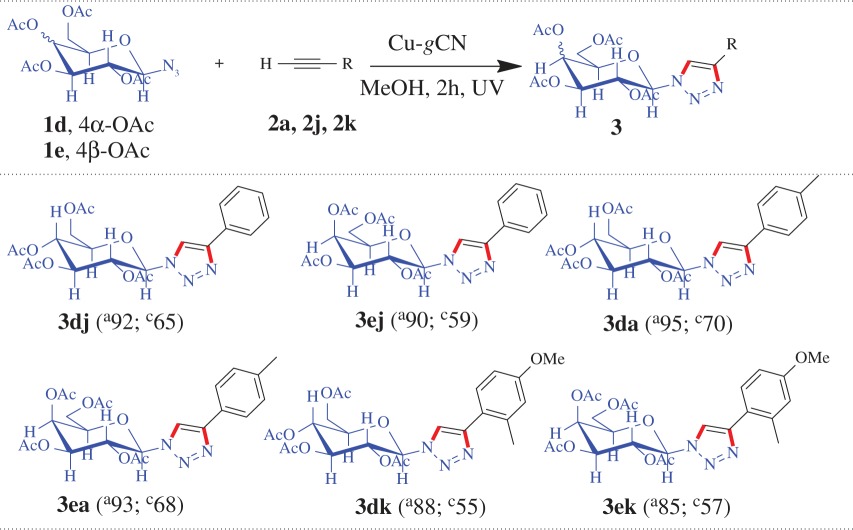

^i^Reaction conditions: sugar azide (373 mg, 1.0 mmol), alkyne (1.0 mmol), MeOH (4 ml), Cu-*g*CN (5.0 mol%).

^ii^Isolated yields (%), a = UV, c = daylight + Et_3_N (1.0 mmol).

## 4. Discussion

**Scheme 1.**
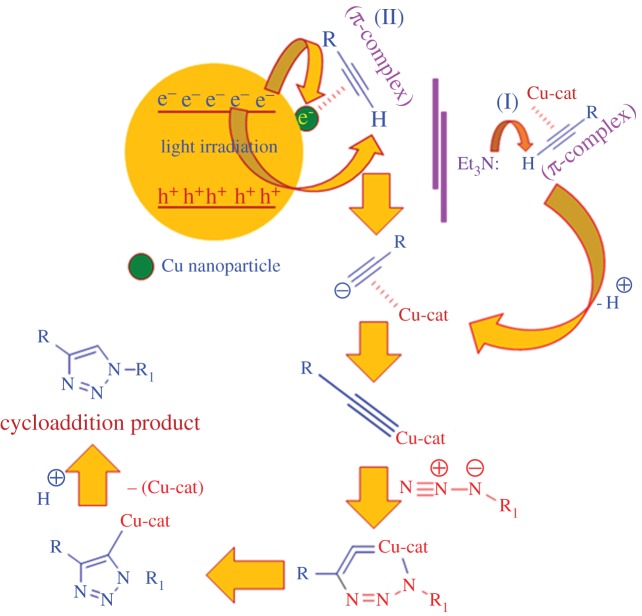
Schematic presentation for the mechanism of triazole formation using Cu-*g*CN composite in the presence of Et_3_N (I) and in the presence of conduction band electron (II).

In general, alkyne–azide cycloaddition process needs a basic medium to initiate the reaction cycle. The mechanistic pathway for the copper-catalysed cycloaddition reaction for triazole synthesis is illustrated in scheme 1 (I), where a π-complex has been formed between the copper and the alkyne molecule followed by the deprotonation of the alkyne molecule, under basic condition, with the formation of copper-acetylidine complex. In the presence of azide molecule, the copper-acetylidine forms a couple of intermediate complexes which subsequently forms the 1,2,3-triazole through protonation along with the elimination of the catalyst. *g*CN is composed of carbon and nitrogen with a unique framework of tri*-s-*triazine linked by tertiary amines, which makes it a promising photocatalyst with a medium band gap. The photocatalytic performance originates from an electron transition from the valence band (VB) populated by N-*2p* orbital to the CB formed by C-*2p* orbital. The photonic effect on the Cu-*g*CN composite catalyst for the cycloaddition reaction is illustrated in scheme 1 (II) where we have already noted that a superior yield has been obtained for all cycloaddition reactions under UV radiation. The photon energy from the ultraviolet source, deep UV (UV-C), is within the range of 6.53–4.43 eV (considering the wavelengths between 190 and 280 nm) and which is the sufficient amount of energy to transfer an electron from the VB to the CB of the *g*CN support (considering the band gap of *g*CN is 2.52 eV, as obtained from the electronic supplementary material, figure S1*a*). The CB electrons have a dual role for the cycloaddition reaction: (i) increasing the charge density of the copper nanoparticles, which ultimately strengthens the metal–alkyne π-complex and lowers the *p*Ka value of the complex, and (ii) also acting as a scavenger for the terminal hydrogen of the alkyne molecule, which leads to the formation of the copper acetylide complex.

The increase of charge density on the copper particles can be explained in the light of Mott–Schottky heterojunction formation. In this work, *g*CN acts as a photoactive support material for the copper nanoparticles. The redox potential of the CB and VB for *g*CN is located at −1.3 eV and +1.4 eV versus NHE, respectively [39], where the work function of the copper nanoparticle is also located [20]. The metal nanoparticles on *g*CN semiconductor form a Mott–Schottky heterojunction and during photon irradiation, some of the CB electron migrates to copper nanoparticles, to create a Schottky barrier, as it matches with the energy level of *g*CN and increases the charge density of the metal. The current reaction has been performed under continuous photon irradiation condition and the possibility of recombination mechanism between electron and hole can be ruled out. It is also important to mention that the proportion of copper nanoparticles is less as compared with *g*CN (5 wt% of Cu), so the majority fraction of the electrons are expected to participate for the deprotonation mechanism of the alkyne molecule.

When the reaction was carried out under UV irradiation in the presence of triethylamine, the base molecule acts as a ‘hole-trap’ species, which interacts with the hole, generated at the VB of *g*CN, through electrostatic attractive force. Spectroscopic evidence supports the widening of the band gap of *g*CN due to the addition of triethylamine. The electronic supplementary material, figure S1A, shows the *g*CN with the band gap of 2.52 eV and an increased band gap of 2.62 eV in the presence of triethylamine. The increased band gap could be the reason for a slight deactivation of the reaction as compared with the UV radiation alone, figure 3*a*,*b*.

We also found that the daylight has a prominent effect on the title reaction as the photon energy value of daylight is in between 3.26 and 1.59 eV (considering all the visible wavelength range from 380 to 780 nm). This amount of photon energy is sufficient to facilitate the electron migration from the VB to the CB of the Cu-*g*CN system. As the daylight has lower photonic energy than the UV, a minimum activation of *g*CN and consequently fewer photo-generated hot electrons can be expected in the CB following the similar mechanistic pathway for the reaction, as mentioned above, with a lower amount of product formation (yield percentage).

**Scheme 2.**
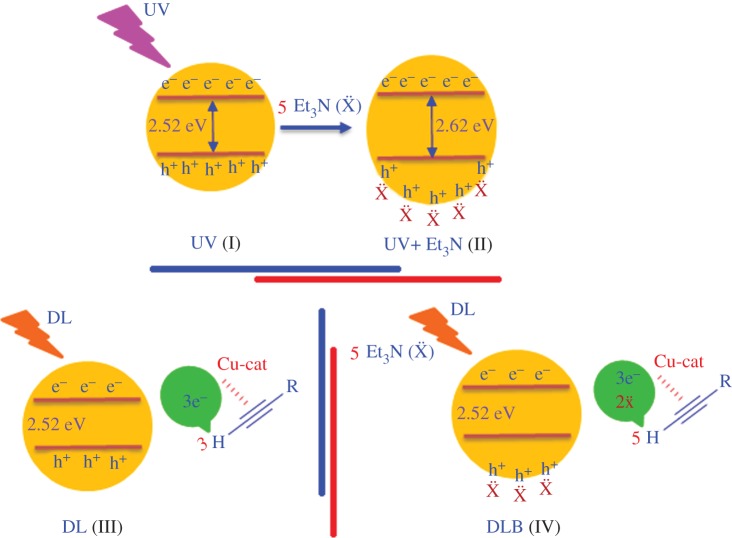
The schematic diagram explains the negative effect on yield formation due to the presence of base under UV irradiation condition. In the scheme, (I) and (II) represent the band gap of *g*CN under UV irradiation and under UV irradiation in the presence of Et_3_N, respectively (derived from the UV-visible spectra, electronic supplementary material figure S1*a*). The cartoon, (III) and (IV) denote the reaction under daylight condition in the absence and presence of Et3N, respectively. To elucidate the process, we have taken the examples where 5e^−^, 5 h^+^ and 5 Et_3_N molecules are involved.

But when we compared the amount of product formation between ‘daylight in the absence of base’ (DL) and ‘daylight in the presence of base’ (DLB), we found that the DLB condition produced higher yield than the DL condition, which is contrary to the result obtained from the ‘UV alone’ and ‘UV in the presence of Et_3_N’ systems. This can be explained as follows: under DLB conditions, the widening of band-gap factor can be neglected, what we have considered for the ‘UV in the presence of Et_3_N’ system, scheme 2 (I and II), as fewer holes have been generated, scheme 2 (III). Under DL conditions, only the photo-generated electrons participated for the deprotonation of the alkyne molecule and Schottky barrier formation mechanism but under DLB condition, along with the above mechanism, the additional base molecule also has the contribution for the deprotonation mechanism, scheme 2 (IV), as the amount of base concentration remains the same when the reactions were performed under both ‘UV in the presence of Et_3_N’ and DLB condition. The schematic diagram (scheme 2) nicely described the above mechanism where 5e^−^, 5 h^+^ and 5Et_3_N molecules have been taken as an example to elucidate the overall process.

**Figure 5.**
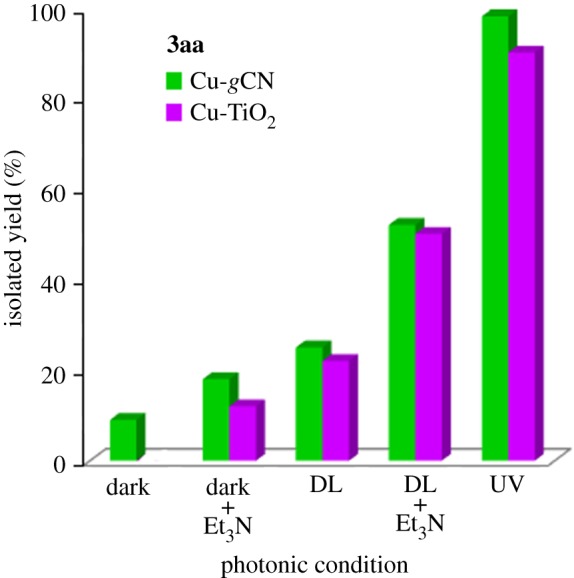
A comparative study between Cu-*g*CN and Cu-TiO_2_.

The current reaction has also been performed under dark conditions in the presence and absence of base, figure 3(e and f) and table 1 (**3aa**). Under the dark condition, the triethylamine showed its own performance for the reaction through deprotonation mechanism. Little activation has also been observed when the reaction was performed under dark conditions in the absence of base molecule, which is due to the presence of amine group in the *g*CN structure, as confirmed by infrared spectroscopy analysis (electronic supplementary material, figure S1*b*), that can scavenge the proton from the alkyne molecule for the copper–acetylidine complex formation. In our previous study, we showed that amine group from aniline is also responsible for the deprotonation mechanism for the azide–alkyne cycloaddition reaction [33]. To confirm the above observation, we have selected titanium dioxide based system, a photoactive material [40], loaded with 5 wt% copper (Cu-TiO_2_) and used it for the title reaction under similar conditions to the Cu-*g*CN system. The comparative results show that both *g*CN and TiO_2_ act as photoactive support material for the cycloaddition reaction, figure 5 (**3aa**). Unlike Cu-gCN, the copper-titanium system acts as a passive material under dark conditions in the absence of base as no suitable moiety is present in the system to facilitate the deprotonation mechanism for scavenge terminal proton of the alkyne. It is also important to mention that TiO_2_ is a higher band gap material [41] than *g*CN and this is reflected through the amount of yield formation (figure 5).

## 5. Conclusion

In this report, we demonstrate that the composite of Cu-*g*CN performs as an effective material for the cycloaddition reaction between azide and alkyne molecules under UV radiation conditions where copper nanoparticles serve as catalyst and the photoactive support material, *g*CN, plays the role of a promoter (as it behaves as a passive material for the cycloaddition reaction alone, both under illuminated and dark conditions). The UV light induced generation of hot electron (CB electron) from the Cu-*g*CN system has dual role for the cycloaddition reaction; firstly, it increases the electron density of the copper particles, which can coordinate with the alkyne molecule to form a metal–alkyne π-complex and make the terminal hydrogen more facile for deprotonation. Secondly, the hot electron scavenges the terminal hydrogen to form the copper acetylide complex. By comparing the yield of the triazole products, formed under different photonic conditions using Cu-*g*CN system, we found that the highest yield has been achieved under UV environment. It is also important to mention that we do not have any evidence of copper leaching during the study on the recycling performance and also due to the photocorrosion effect of the catalyst (supporting documents). This work not only provided an efficient light-induced strategy for organic synthesis, but also offered a new mechanism, which could further be applied for designing new and improved catalyst that can eliminate the hazardous chemicals necessary for the reaction under conventional protocol.

## Supplementary Material

Electronic supplementary materialUV, IR, NMR spectra etc.

## Data accessibility

The datasets supporting this article have been uploaded as part of the electronic supplementary material.
