# Supplementary material for: Carbon nitride supported copper nanoparticles: light-induced electronic effect of the support for triazole synthesis
Source: R Soc Open Sci. 2016 Nov 16;3(11):160580. doi: 10.1098/rsos.160580 (PMC5180146; doi:10.1098/rsos.160580)
Supplement: Electronic supplementary materialUV, IR, NMR spectra etc. [file rsos160580supp1.docx]

**Carbon nitride supported copper nanoparticles: Light induced electronic effect of the support for triazole synthesis**

**Debkumar Nandi, Abu Taher, Rafique Ul Islam, Samarjeet Siwal, Meenakshi Choudhary, Kaushik Mallick***

Department of Chemistry, University of Johannesburg, P.O. Box: 524, Auckland Park 2006, South Africa.

*List of contents*

| **General Considerations** | **S1** |
| --- | --- |
| **UV and IR spectrum of *g*CN** | **S2-S3** |
| **Recycling performance of a catalyst** | **S3-S4** |
| **Characterization of Products** | **S5-S12** |
| **NMR Spectra** | **S13-S29** |

**GENERAL CONSIDERATIONS:**

Solvents were distilled from appropriate drying agent prior to use. Commercially available reagents were used without further purification unless otherwise stated. ^1^H and ^13^C NMR spectra were recorded on a Bruker AVANCE^III^-400 spectrometer.^1^H NMR (400 MHz) and ^13^C NMR (100 MHz) were registered in CDCl_3_ as solvent and tetramethylsilane (TMS) as an internal standard. Chemical shifts are reported in δ units (ppm). All coupling constants (*J*) are reported in hertz (Hz). HRMS was carried out on a sector field mass spectrometer.

**
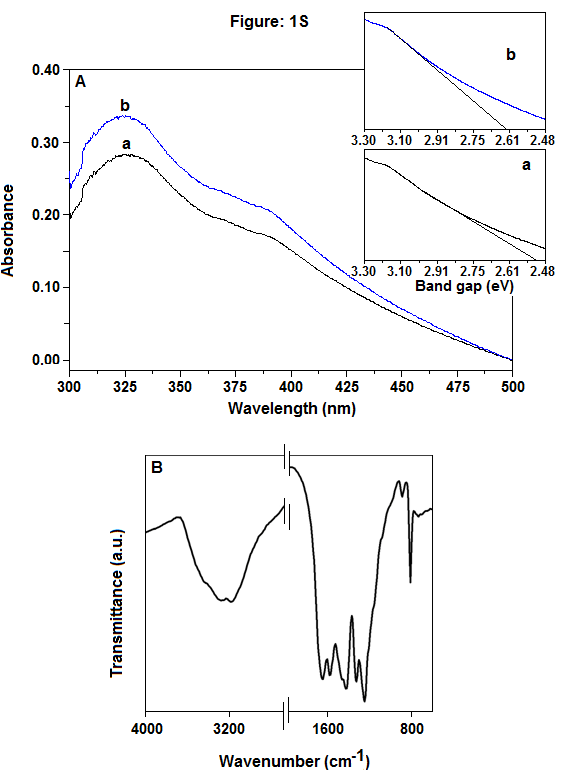
**

**Figure 1S:** (A) UV-visible spectroscopic analysis shows the *g*CN with the band gap of 2.52 eV (a) and the *g*CN in the presence of triethylamine with an increased band gap of 2.62 eV (b).

(B) The Fourier transform infrared spectroscopy measurement of Cu-*g*CN revealed a typical molecular structure of *g*CN. The vibration bands at 1642, 1566 and 1417 cm^−1^ were assigned to typical stretching vibration modes of heptazine***-***derived repeating units which agree with the FTIR analysis for the g-CN [1]. The intense band at 812 cm^−1^ represented the out-of-plane bending vibration and the typical characteristic of heptazine rings. The bands at 1315 and 1241 cm^−1^ correspond to stretching vibration of connected units of C-N(-C)(-C) (full condensation) or C-NH-C (partial condensation). The broad band with a doublet between 3100-3300 cm^−1^ corresponds to the stretching modes of –NH_2_ or =NH groups, which are uncondensed amine groups.

**Recycling performance of a catalyst:**

The recycling performance of a catalyst for the heterogeneous catalysis reaction is an important parameter from the industrial point of view.

The reaction between 1-(azidomethyl)-4-methylbenzene (**1a**) and 1-ethynyl-4-methylbenzene (**2a**) were chosen as the substrates for the recyclability study of the cycloaddition reaction, under UV-light and other optimized reaction conditions, to obtain the desired product, 1-(4-methylbenzyl)-4-p-tolyl-1H-1,2,3-triazole (**3aa**).

To perform the recyclability study, we have increased the amount for all the reactants, reagents and the catalyst by a factor of ten. A yield of 98% of the targeted the product **3aa** was obtained after the first cycle, whereas at the end of the fifth cycle, a slight deactivation of the reaction has been noticed with the yield of 89% of **3aa**. At the end of each cycle, the catalyst was recovered by filtration technique then washed with methanol for several times followed by drying in air and was reused for the next cycle. The graphical representation (Figure 2S) shows the yield percentage of the product (**3aa**) as a function of ‘number of cycles’ where it could be concluded that the used material was also behave as an active catalyst without significant loss of catalytic performance.

The filtrate of every cycle was tested with ICP-MS (Inductively coupled plasma mass spectrometry) analysis and there was no evidence of copper leaching has been detected by the instrument.


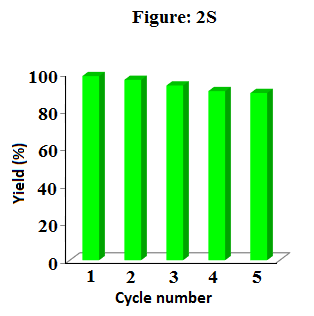


**Figure 2S**: Graphical presentation of reusability study for 5 catalytic cycles.

**CHARACTERIZATION OF CYCLOADDUCTS:**

**1-(4-methylbenzyl)-4-p-tolyl-1H-1,2,3-triazole** (**3aa**): ^2^ White solid, (yield: for Cu-*g*CN UV: 258 mg, 98%; UV+ Et_3_N: 242 mg, 92%; Daylight + Et_3_N: 137 mg 52%; Daylight: 66 mg 25%; Dark + Et_3_N: 47 mg 18%; Dark: 24 mg 9%; for Cu-TiO_2_ UV: 237 mg, 90%; UV+ Et_3_N: 210 mg, 80%; Daylight + Et_3_N: 132 mg 50%; Daylight: 58 mg 22%; Dark + Et_3_N: 32 mg 12%; Dark: 0 mg, 0%; cycle 1: 2.58 g, 98%; cycle 2: 2.52 g, 96, cycle 3: 2.45 g 93%, cycle 4: 2.37 g, 90% and cycle 5: 2.34 g, 89%) mp: 123-125^o^C. Synthesized following the general procedure from 1-(azidomethyl)-4-methylbenzene **1a** (0.13 mL, 1.0 mmol), 1-ethynyl-4-methylbenzene **2a** (116 mg, 1.0 mmol).

**^1^H NMR (400 MHz, CDCl_3_):** δ 2.33 (s, 3H), 2.34 (s, 3H), 5.47 (s, 2H), 7.11-7.19 (m, 6H), 7.59 (s, 1H), 7.66 (d, *J*= 8.0 Hz, 2H).

**^13^C NMR (100 MHz, CDCl_3_):** δ 21.0, 21.1, 53.8, 119.0, 125.5 (2C), 127.7, 127.9 (2C), 129.3 (2C), 129.6 (2C), 131.6, 137.8, 138.5, 148.0.

**HRMS:** (ESI) m/z [M]^+^ calcd for C_17_H_17_N_3_ 263.1422, found 263.1421.

**1-(4-methylbenzyl)-4-propyl-1*H*-1,2,3-triazole** (**3ab**): Pale yellow liquid, (yield: UV: 205 mg, 95%; UV+ Et_3_N: 196 mg, 91%; Daylight+ Et_3_N: 127 mg, 59%). Synthesized following the general procedure from 1-(azidomethyl)-4-methylbenzene **1a** (0.13 mL, 1.0 mmol), pent-1-yne **2b** (68 mg, 1.0 mmol).

**^1^H NMR (400 MHz, CDCl_3_):** δ 0.89 (t, *J*= 7.2 Hz, 3H), 1.36-1.30 (m, 2H), 1.57-1.61 (m, 2H), 2.33 (s, 3H), 2.65 (t, *J*= 7.8 Hz, 2H ), 5.42 (s, 2H), 7.13-7.14 (m, 5H);

**^13^C NMR (100 MHz, CDCl_3_**): δ 13.8, 21.1, 22.3, 31.5, 53.8, 115.1, 127.9 (2C), 129.3, 129.7 (2C), 131.9, 138.47.

**HRMS:** (ESI) m/z [M]^+^ calcd for C_13_H_17_N_3_ 215.1422, found 215.1422.

**1-(4-methylbenzyl)-4-(4-nitrophenyl)-1H-1,2,3-triazole** (**3ac**): Light yellow solid, (yield: UV: 262 mg, 89%; UV+ Et_3_N: 244 mg, 83%; Daylight+ Et_3_N: 180 mg, 61%), mp: 200-201^o^C. Synthesized following the general procedure from 1-(azidomethyl)-4-methylbenzene **1a** (0.13 mL, 1.0 mmol), 1-ethynyl-4-nitrobenzene **2c** (147 mg, 1.0 mmol).

**^1^H NMR (400 MHz, CDCl_3_):** δ 2.34 (s, 3H), 5.53 (s, 2H), 7.19-7.20 (m, 4H), 7.77 (s, 1H), 7.93 (d, J= 8.4 Hz, 2H), 8.21 (d, J= 8.8 Hz, 2H).

**^13^C NMR (100 MHz, CDCl_3_):** δ 21.1, 54.2, 120.8, 124.2 (2C), 126.1 (2C), 128.2 (2C), 129.9 (2C), 131.1, 136.9, 139.0, 145.9, 1473.

**HRMS:** (ESI) m/z [M]^+^ calcd for C_16_H_14_N_4_O_2_ 294.1117, found 294.1120.

**Methyl 1-benzyl-1*H*-1,2,3-triazole-4-carboxylate** (**3bd**): ^2^ White solid, (yield: UV: 200 mg, 92%; UV+ Et_3_N: 193 mg, 89%; Daylight+ Et_3_N: 140 mg, 65%), mp: 114-115^o^C. Synthesized following the general procedure from azidomethylbenzene **1b** (133 mg, 1.0 mmol), methyl propiolate **2d** (84 mg, 1.0 mmol).

**^1^H NMR (400 MHz, CDCl_3_):** δ 3.80 (s, 3H), 5.50 (s, 2H), 7.18-7.20 (m, 2H), 7.25-7.28 (m, 3H), 7.99 (s, 1H).

**^13^C NMR (100 MHz, CDCl_3_):** δ 51.8, 54.1, 127.3, 128.0 (2C), 128.8, 129.0 (2C), 133.6, 139.9, 160.8.

**HRMS:** (ESI) m/z [M]^+^ calcd for C_11_H_11_N_3_O_2_ 217.0851, found 294.0849.

 **Ethyl 1-benzyl-1*H*-1,2,3-triazole-4-carboxylate** (**3be**): ^2^ Pale yellow solid, (yield: UV: 217 mg, 94%; Daylight+ Et_3_N: 134 mg, 58%), mp: 92-93^o^C. Synthesized following the general procedure from azidomethylbenzene **1b** (133 mg, 1.0 mmol), ethyl propiolate **2e** (98 mg, 1.0 mmol).

**^1^H NMR (400 MHz, CDCl_3_):** δ 1.32 (t, *J* = 7.0 Hz, 3H), 4.33 (q, *J* = 7.2 Hz, 2H), 5.53 (s, 2H), 7.22-7.25(m, 2H), 7.31-7.35 (m, 3H), 7.96 (s, 1H).

**^13^C NMR (100 MHz, CDCl_3_):** δ 14.1, 54.3, 61.1, 127.2, 128.1 (2C), 128.9, 129.1 (2C), 133.7, 140.4, 160.5.

**HRMS:** (ESI) m/z [M]^+^ calcd for C_12_H_13_N_3_O_2_ 231.1008, found 231.1011.

**1-benzyl-4-propyl-1H-1,2,3-triazole** (**3bb**): ^3^ Light yellow liquid, (yield: UV: 183 mg, 91%; Daylight+ Et_3_N: 103 mg, 51%). Synthesized following the general procedure azidomethylbenzene **1b** (133 mg, 1.0 mmol), methyl pent-1-yne **2b** (68 mg, 1.0 mmol).

**^1^H NMR (400 MHz, CDCl_3_):** δ 0.89 (t, *J* = 7.2 Hz, 3H), 1.61 (q, *J* = 7.6 Hz, 2H,), 2.61 (t, *J* = 7.2 Hz, 2H), 5.43 (s, 2H), 7.18-7.21 (m, 3H), 7.27-7.32 (m, 3H).

**^13^C NMR (100 MHz, CDCl_3_):** δ 13.6, 22.5, 27.5, 53.8, 120.6, 127.8 (2C), 128.4, 128.9 (2C), 134.8, 148.5.

**HRMS:** (ESI) m/z [M]^+^ calcd for C_12_H_15_N_3_ 201.1266, found 201.1268.

 **1-Benzyl-4-butyl-1*H*-1,2,3-triazole** (**3bf**): ^3^ Yellow liquid, (yield: UV: 194 mg, 90%; Daylight+ Et_3_N: 120 mg, 56%). Synthesized following the general procedure from azidomethylbenzene **1b** (133 mg, 1.0 mmol), hex-1-yne **2f** (82 mg, 1.0 mmol).

**^1^H NMR (400 MHz, CDCl_3_):** δ 0.81 (t, *J* = 7.2 Hz, 3H), 1.26 (m, 2H), 1.51 (m, 2H), 2.58 (t, *J* = 7.8 Hz, 2H), 5.38 (s, 2H), 7.13-7.16 (m, 3H), 7.21-7.24 (m, 3H).

**^13^C NMR (100 MHz, CDCl_3_):** δ 13.5, 21.9, 25.0, 31.2, 53.5, 120.4, 127.6 (2C), 128.2, 128.6 (2C), 134.8, 148.4.

**HRMS:** (ESI) m/z [M]^+^ calcd for C_13_H_17_N_3_ 215.1422, found 215.1419.

**4-((1-benzyl-1*H*-1,2,3-triazol-4-yl)methoxy)-6-methyl-2H-pyran-2-one** (**3bg**): White solid, (yield: UV: 253 mg, 85%; Daylight+ Et_3_N: 160 mg, 54%), mp: 133-134^o^C. Synthesized following the general procedure from azidomethylbenzene **1b** (133 mg, 1.0 mmol), 6-methyl-4-(prop-2-ynyloxy)-2H-pyran-2-one **2g** (164 mg, 1.0 mmol).

**^1^H NMR (400 MHz, CDCl_3_):** δ 2.11 (s, 3H), 5.05 (s, 2H), 5.47 (d, *J*= 2.0 Hz, 1H), 5.50 (s, 2H), 5.72 (d, *J*= 0.8 Hz, 1H), 7.23-7.25 (m, 2H), 7.31-7.34 (m, 3H), 7.57 (s, 1H).

**^13^C NMR (100 MHz, CDCl_3_):** δ 19.7, 54.2, 62.0, 88.2, 100.2, 123.3, 128.1 (2C), 128.8, 129.1 (2C), 129.2, 134.1, 162.2, 164.5, 169.7.

**HRMS:** (ESI) m/z [M]^+^ calcd for C_16_H_15_N_3_O_3_ 297.1113, found 297.1115.

 **1-(1-benzyl-1*H*-1,2,3-triazol-4-yl)cyclopentanol** (**3bh**): ^4^ White solid, (yield: UV: 214 mg, 88%; Daylight+ Et_3_N: 129 mg, 53%), mp: 71-72^o^C. Synthesized following the general procedure from azidomethylbenzene **1b** (133 mg, 1.0 mmol), 1-ethynylcyclopentanol **2h** (110 mg, 1.0 mmol).

**^1^H NMR (400 MHz, CDCl_3_):** δ 1.68-1.72 (m, 2H), 1.83-2.02 (m, 6H), 3.33 (s, 1H), 5.39 (s, 2H), 7.17-7.20 (m, 2H), 7.27-7.30 (m, 3H), 7.38 (s, 1H).

**^13^C NMR (100 MHz, CDCl_3_):** δ 23.4 (2C), 40.9 (2C), 53.8, 78.6, 119.8, 127.9 (2C), 128.4, 128.8 (2C), 134.5, 154.6.

**HRMS:** (ESI) m/z [M]^+^ calcd for C_14_H_17_N_3_O 243.1372, found 243.1373.

**1-(1-benzyl-1*H*-1,2,3-triazol-4-yl)cyclohexanol** (**3bi**): ^4^ White solid, (yield: UV: 213 mg, 83%; Daylight+ Et_3_N: 152 mg, 59%), mp: 100-101^o^C. Synthesized following the general procedure from azidomethylbenzene **1b** (133 mg, 1.0 mmol), 1-ethynylcyclohexanol **2i** (124 mg, 1.0 mmol).

**^1^H NMR (400 MHz, CDCl_3_):** δ 1.23-1.31 (m, 1H), 1.43-1.56 (m, 3H), 1.62-1.72 (m, 2H), 1.78-1.92 (m, 4H), 3.02 (s, 1H), 5.42 (s, 2H), 7.19-7.21 (m, 2H), 7.29-7.31 (m, 3H), 7.35 (s, 1H).

**^13^C NMR (100 MHz, CDCl_3_):** δ 21.8 (2C), 25.2, 37.9 (2C), 53.9, 69.3, 119.5, 127.9 (2C), 128.5, 128.9 (2C), 134.4, 156.0.

**HRMS:** (ESI) m/z [M]^+^ calcd for C_15_H_19_N_3_O 257.1528, found 257.1530.

 **1-(2-Bromobenzyl)-4-phenyl-1*H*-1,2,3-triazole** (**3cj**): ^2^ White solid, (yield: UV: 280 mg, 89%; Daylight+ Et_3_N: 151 mg, 48%), mp: 110-111^o^C. Synthesized following the general procedure from 1-(azidomethyl)-2-bromobenzene **1c** (212 mg, 1.0 mmol), phnylacetelene **2j** (102 mg, 1.0 mmol).

**^1^H NMR (400 MHz, CDCl_3_):** δ 5.60 (s, 2H), 7.08 (dd, *J*=7.6, 1.6 Hz, 1H), 7.18 (dt, *J*=7.6, 1.6 Hz, 1H), 7.22-7.33 (m, 2H), 7.35 (t, *J*=7.6, 2H), 7.55 (dd, *J*=7.2, 1 Hz, 1H) 7.61-7.79 (m, 3H). **^13^C NMR (100 MHz, CDCl_3_):** δ 53.6, 119.8, 123.1, 125.5 (2C), 127.9, 128.0, 128.6, 129.9 (2C), 130.1, 130.3, 132.9, 134.0, 147.8.

**HRMS:** (ESI) m/z [M]^+^ calcd for C_15_H_12_BrN_3_ 313.0215, found 313.0212.

**4-Phenyl-1-(2,3,4,6-tetra-*O*-acetyl-β-D-glucopyranosyl)-1*H*-1,2,3-triazole** (**3dj**): ^5^ White solid, (yield: UV: 432 mg, 92%; Daylight+ Et_3_N: 309 mg, 65%), mp: 211-212^o^C. Synthesized following the general procedure from 1-azido-2,3,4,6-tetra-*O*-acetyl-β-D-glucopyranose **1d** (373 mg, 1.0 mmol), phnylacetelene **2j** (102 mg, 1.0 mmol).

**^1^H NMR (400 MHz, CDCl_3_):** δ1.87 (s, 3H), 1.99 (s, 3H), 2.01 (s, 3H), 2.22 (s, 3H), 4.13-4.18 (m, 2H), 4.21-4.26 (m, 1H), 5.26 (dd, *J*= 10.2, 3.4 Hz, 1H), 5.55 (d, *J*= 2.8 Hz, 1H), 5.61 (t, *J*= 9.8 Hz, 1H), 5.88 (d, *J*= 9.2 Hz, 1H), 7.30-7.34 (m, 1H), 7.38-7.42 (m, 2H), 7.82-7.84 (m, 2H), 8.03 (s, 1H).

**^13^C NMR (100 MHz, CDCl_3_):** δ 20.2, 20.4, 20.5, 20.6, 61.2, 66.9, 68.0, 70.8, 74.0, 86.2, 117.8, 125.8 (2C), 128.4, 128.8 (2C), 129.9, 148.4, 169.1, 169.7, 169.9, 170.3.

**HRMS:** (ESI) m/z [M]^+^ calcd for C_22_H_25_N_3_O_9_ 475.1591, found 475.1589.

**4-Phenyl-1-(2,3,4,6-tetra-*O*-acetyl-β-D-galactopyranosyl)-1*H*-1,2,3-triazole** (**3ej**): ^5^ White solid, (yield: UV: 428 mg, 90%; Daylight+ Et_3_N: 280 mg, 59%), mp: 208-209^o^C. Synthesized following the general procedure from 1-azido-2,3,4,6-tetra-*O*-acetyl-β-D-galactopyranose **1e** (373 mg, 1.0 mmol), phnylacetelene **2j** (102 mg, 1.0 mmol).

**^1^H NMR (400 MHz, CDCl_3_):** δ1.87 (s, 3H), 1.99 (s, 3H), 2.02 (s, 3H), 2.22 (s, 3H), 4.01-4.17 (m, 2H), 4.20-4.23 (m, 1H), 5.24 (dd, *J*= 10.2, 3.4 Hz, 1H), 5.54 (d, *J*= 3.2, 1H), 5.61 (t, *J*= 9.8 Hz, 1H), 5.86 (d, *J*= 9.6 Hz, 1H), 6.94 (d, *J*= 8.8 Hz, 3H), 7.75 (d, *J*= 8.8 Hz, 2H), 7.93 (s, 1H). **^13^C NMR (100 MHz, CDCl_3_):** δ 20.6, 20.9, 21.0, 21.1, 61.6, 67.3, 68.1, 71.2, 74.4, 86.7, 114.6 (2C), 117.3, 123.1, 127.6 (2C), 133.8, 148.7, 169.5, 170.2, 170.4, 170.7.

**HRMS:** (ESI) m/z [M]^+^ calcd for C_22_H_25_N_3_O_9_ 475.1591, found 475.1590.

**4-p-tolyl-1-(2,3,4,6-tetra-*O*-acetyl-β-D--glucopyranosyl)-1*H*-1,2,3-triazole** (**3da**): ^6^ White solid, (yield: UV: 467 mg, 95%; Daylight+ Et_3_N: 342 mg, 70%), mp: 198-199^o^C. Synthesized following the general procedure from 1-azido-2,3,4,6-tetra-*O*-acetyl-β-D-glucopyranose **1d** (373 mg, 1.0 mmol), 1-ethynyl-4-methylbenzene **2a** (116 mg, 1.0 mmol).

**^1^H NMR (400 MHz, CDCl_3_):** δ1.85 (s, 3H), 2.01 (s, 3H), 2.05 (s, 3H), 2.06 (s, 3H), 2.35 (s, 3H), 3.99-4.03 (m, 1H) 4.13 (dd, *J*= 12.4, 1.6 Hz, 1H), 4.30 (dd, *J*= 12.6, 5.0 Hz, 1H), 5.24 (t, *J*= 9.6 Hz, 1H), 5.43 (t, *J*= 9.6 Hz, 1H), 5.47 (t, *J*= 9.6 Hz, 1H), 5.91 (d, *J*= 8.0 Hz, 1H) 7.21 (d, *J*= 7.6 Hz, 2H), 7.70 (d, *J*= 8.0Hz, 2H), 7.93 (s, 1H).

**^13^C NMR (100 MHz, CDCl_3_):** δ 20.1 (2C), 20.5, 20.6, 21.3, 61.6, 67.8, 70.2, 72.8, 75.1, 85.7, 117.3, 125.8 (2C), 127.1, 129.5 (2C), 138.4, 148.5, 168.9, 169.3, 169.9, 170.4.

**HRMS:** (ESI) m/z [M]^+^ calcd for C_23_H_27_N_3_O_9_ 489.1747, found 489.1749.

**4-p-tolyl-1-(2,3,4,6-tetra-*O*-acetyl-β-D-galactopyranosyl)-1*H*-1,2,3-triazole** (**3ea**): White solid, (yield: UV: 455 mg, 93%; Daylight+ Et_3_N: 333 mg, 68%), mp: 200-201^o^C. Synthesized following the general procedure from 1-azido-2,3,4,6-tetra-*O*-acetyl-β-D-galactopyranose **1e** (373 mg, 1.0 mmol), 1-ethynyl-4-methylbenzene **2a** (116 mg, 1.0 mmol).

**^1^H NMR (400 MHz, CDCl_3_):** δ1.84 (s, 3H), 1.97 (s, 3H), 1.99 (s, 3H), 2.19 (s, 3H), 2.33 (s, 3H), 4.12-4.16 (m, 2H) 4.22-4.24 (m, 1H), 5.25 (dd, *J*= 10.2, 3.4 Hz, 1H), 5.52 (d, *J*= 2.8 Hz, 1H), 5.60 (t, *J*= 9.8 Hz, 1H), 5.88 (d, *J*= 9.2 Hz, 1H), 7.19 (d, *J*= 8.0 Hz, 2H), 7.70 (d, *J*= 8.0Hz, 2H), 7.99 (s, 1H).

**^13^C NMR (100 MHz, CDCl_3_):** δ 20.1(2C), 20.4, 20.4, 21.1, 61.1, 66.9, 67.7, 70.8, 73.9, 86.1, 117.4, 125.7 (2C), 127.1, 129.4 (2C), 138.2, 148.3, 169.0, 169.7, 169.9, 170.2.

**HRMS:** (ESI) m/z [M]^+^ calcd for C_23_H_27_N_3_O_9_ 489.1747, found 489.1746.

**4-(4-methoxy-2-methyl-phenyl)-1-(2,3,4,6-tetra-*O*-acetyl-β-D--glucopyranosyl)-1*H*-1,2,3-triazole** (**3dk**): White solid, (yield: UV: 457 mg, 88%; Daylight+ Et_3_N: 285 mg, 55%), mp: 215-215^o^C. Synthesized following the general procedure from 1-azido-2,3,4,6-tetra-*O*-acetyl-β-D-glucopyranose **1d** (373 mg, 1.0 mmol), 1-ethynyl-4-methoxy-2-methylbenzene **2k** (146 mg, 1.0 mmol).

**^1^H NMR (400 MHz, CDCl_3_):** δ1.97 (s, 6H), 2.01 (s, 6H), 2.36 (s, 3H), 3.76 (s, 3H), 3.99-4.13 (m, 2H), 4.26 (dd, *J*= 12.4, 4.8 Hz, 1H), 5.23 (t, *J*= 1,6 Hz, 1H), 5.40 (t, *J*= 9.4 Hz, 1H), 5.47-5.52 (m, 1H) 5.91 (d, *J*= 9.2, 1H), 6.75 (s, 2H), 7.60-7.62 (m, 1H), 7.80 (s, 1H).

**^13^C NMR (100 MHz, CDCl_3_):** δ 20.1, 20.5, 20.6, 21.0, 21.4, 55.2, 61.6, 67.8, 70.3, 72.7, 75.0, 85.7, 114.4, 116.2, 119.4, 122.0, 130.3, 137.3, 147.5, 159.6, 168.9, 169.4, 169.8, 170.4.

**HRMS:** (ESI) m/z [M]^+^ calcd for C_24_H_29_N_3_O_10_ 519.1853, found 519.1852.

**4-(4-methoxy-2-methyl-phenyl)-1-(2,3,4,6-tetra-*O*-acetyl-β-D-galactopyranosyl)-1*H*-1,2,3-triazole** (**3ek**): White solid, (yield: UV: 441 mg, 85%; Daylight+ Et_3_N: 296 mg, 57%), mp: 212-213^o^C. Synthesized following the general procedure from 1-azido-2,3,4,6-tetra-*O*-acetyl-β-D-galactopyranose **1e** (373 mg, 1.0 mmol), 1-ethynyl-4-methoxy-2-methylbenzene **2k** (146 mg, 1.0 mmol).

**^1^H NMR (400 MHz, CDCl_3_):** δ1.85 (s, 3H), 1.97 (s, 3H), 1.99 (s, 3H), 2.18 (s, 3H) 2.39 (s, 3H), 3.78 (s, 3H), 4.12-4.16 (m, 2H), 4.22-4.26 (m, 1H), 5.26 (dd, *J*= 10.4, 3.2 Hz, 1H), 5.53 (d, *J*= 2.8 Hz, 1H), 5.60 (t, *J*= 10.0 Hz, 1H), 5.88 (d, *J*= 9.2 Hz, 1H) 6.77 (s, 2H), 7.62 (d, *J*= 9.2 Hz, 1 H) 7.81 (s, 1H).

**^13^C NMR (100 MHz, CDCl_3_):** δ 20.1 (2C), 20.4, 20.5, 21.3, 55.1, 61.1, 66.9, 67.8, 70.7, 73.9, 86.2, 111.3, 116.1, 119.4, 122.0, 130.3, 137.3, 147.5, 159.5, 169.0, 169.7, 169.9, 170.2.

**HRMS:** (ESI) m/z [M]^+^ calcd for C_24_H_29_N_3_O_10_ 519.1853, found 519.1856.

**EXPERIMENTAL REFERENCES**:

[1] J. Liu, T. Zhang, Z. Wang, G. Dawson, W. Chen, J. Mater. Chem. 21 (2011) 14398-14401.

[2] (a) R. U. Islam, A. Taher, M. Choudhary, S. Siwal, K. Mallick, Sci. Rep. 5 (2015**)** 9632. (b) R. U. Islam, A. Taher, M. Choudhary, J. M.  Witcomb, K.  Mallick, 
Dalton Trans. 44 (2014) 1341-1349. (c) A. Taher, D. Nandi, R. U. Islam, M. Choudhary, K. Mallick, RSC Advances 5 (2015) 47275-47283. (d) K. Kamata, Y. [Nakagawa,](http://0-fl-www.reaxys.com.ujlink.uj.ac.za/reaxys/secured/paging.do?performed=true&action=get_preparations&databaseId=0&rnd=0.27824762626005306) K.  [Yamaguchi,](http://0-fl-www.reaxys.com.ujlink.uj.ac.za/reaxys/secured/paging.do?performed=true&action=get_preparations&databaseId=0&rnd=0.27824762626005306) N.  [Mizuno,](http://0-fl-www.reaxys.com.ujlink.uj.ac.za/reaxys/secured/paging.do?performed=true&action=get_preparations&databaseId=0&rnd=0.27824762626005306) J. Am. Chem. Soc. 130 (2008**)** 15304-15310.

[3] B. Movassagh, N.  Rezaei, Tetrahedron 70 (2014) 8885-8892.

[4] J. I. Sarmiento-Sanchez, A. Ochoa-Teran, I. A. Rivero, Arkivoc 9 (2011) 177-188.

[5] B. R. Buckley, M. M. P. Figueres, A. N.  Khan, H.  Heaney, Synlett 27 (2016) 51-56.

[6] O. Schwardt, S. Rabbani, M. Hartmann, D. Abgottspon, M. Wittwer, S. Kleeb, A. Zalewski, M. Smiesko, B. Cutting, B. Ernst, Bioorg. Med. Chem. 19 (2011) 6454-6473.


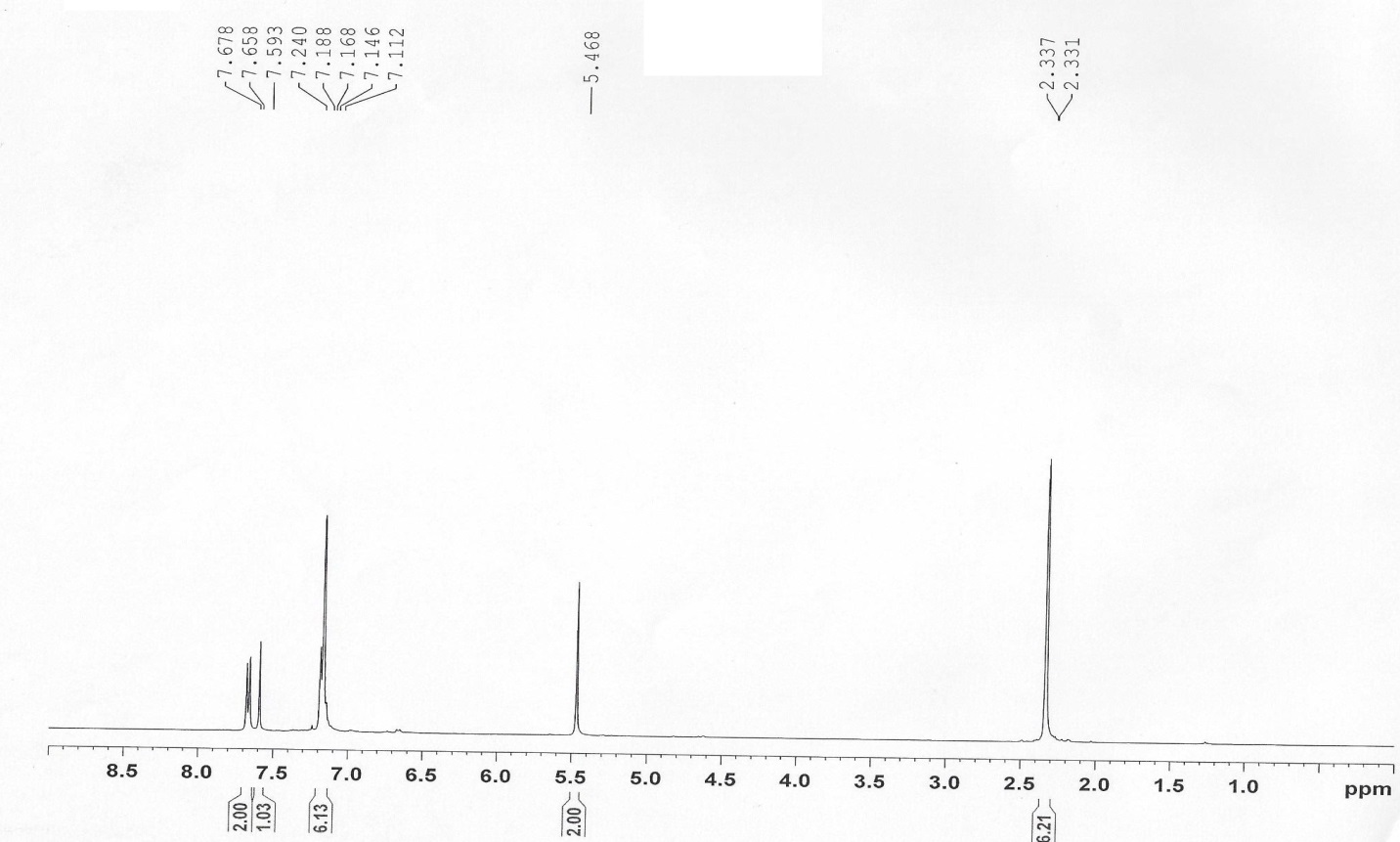


**Figure S1**. ^1^H NMR spectrum of **3aa** in CDCl_3_


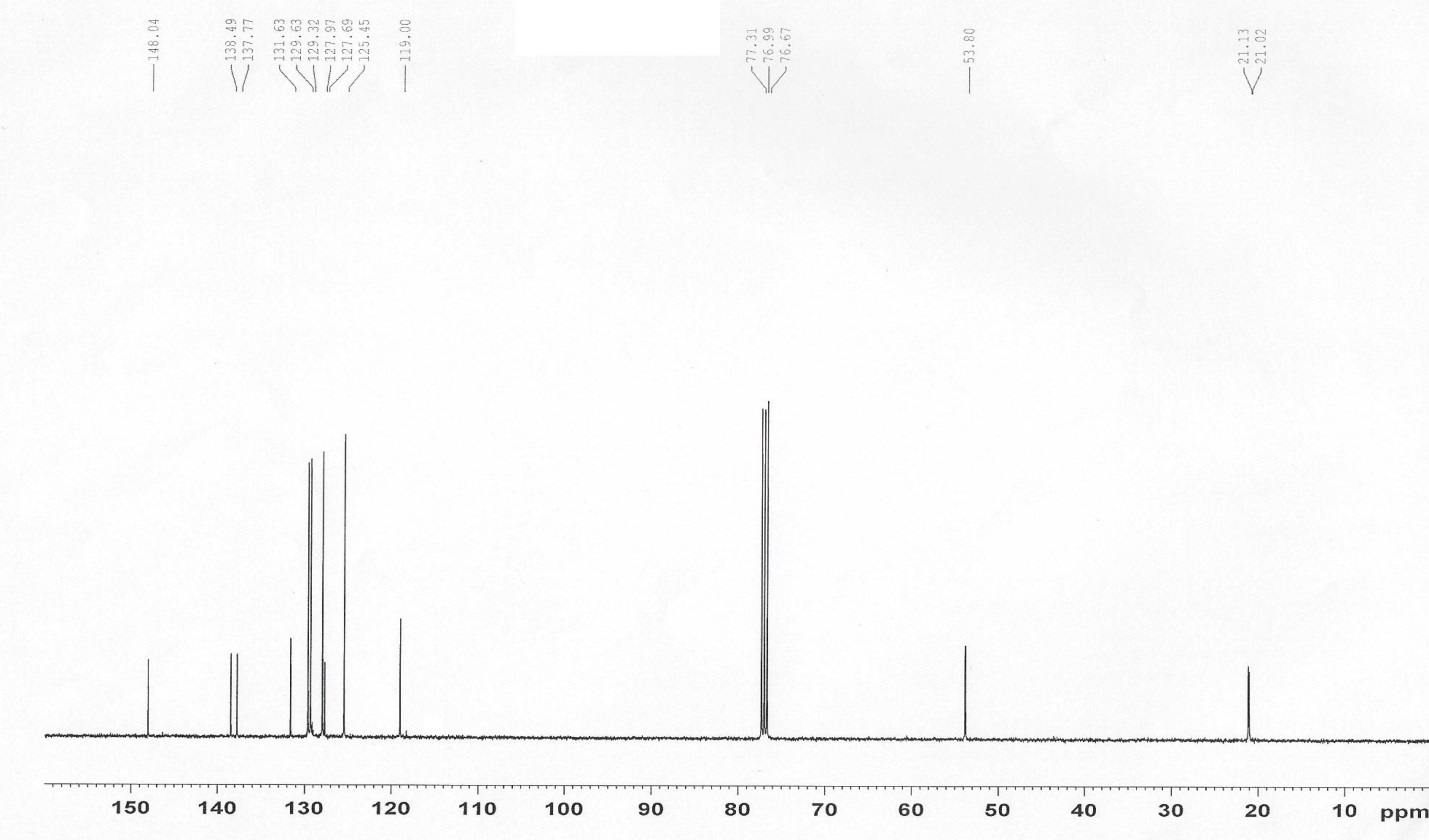


**Figure S2**. ^13^C NMR spectrum of **3aa** in CDCl_3_


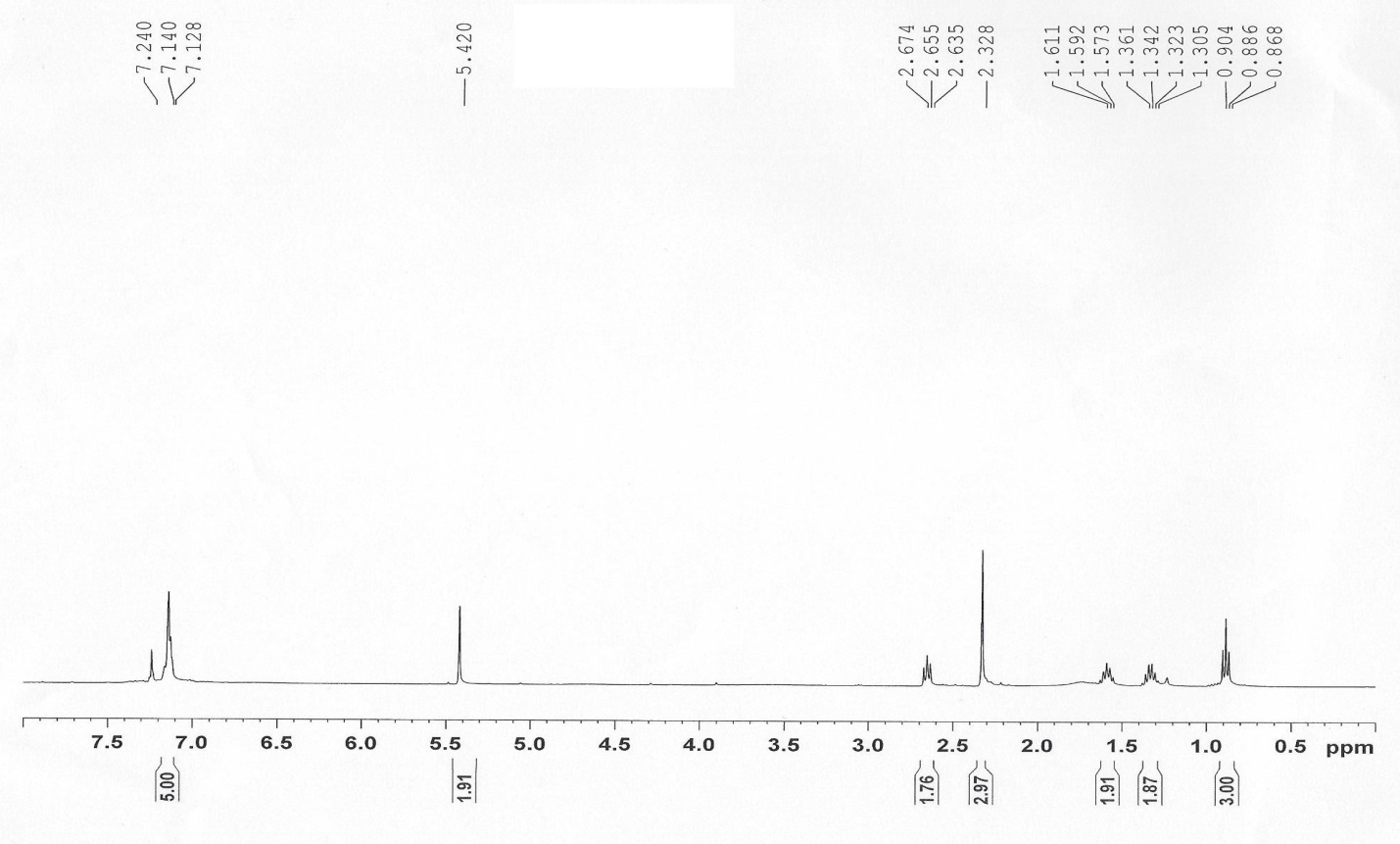


**Figure S3**. ^1^H NMR spectrum of **3ab** in CDCl_3_


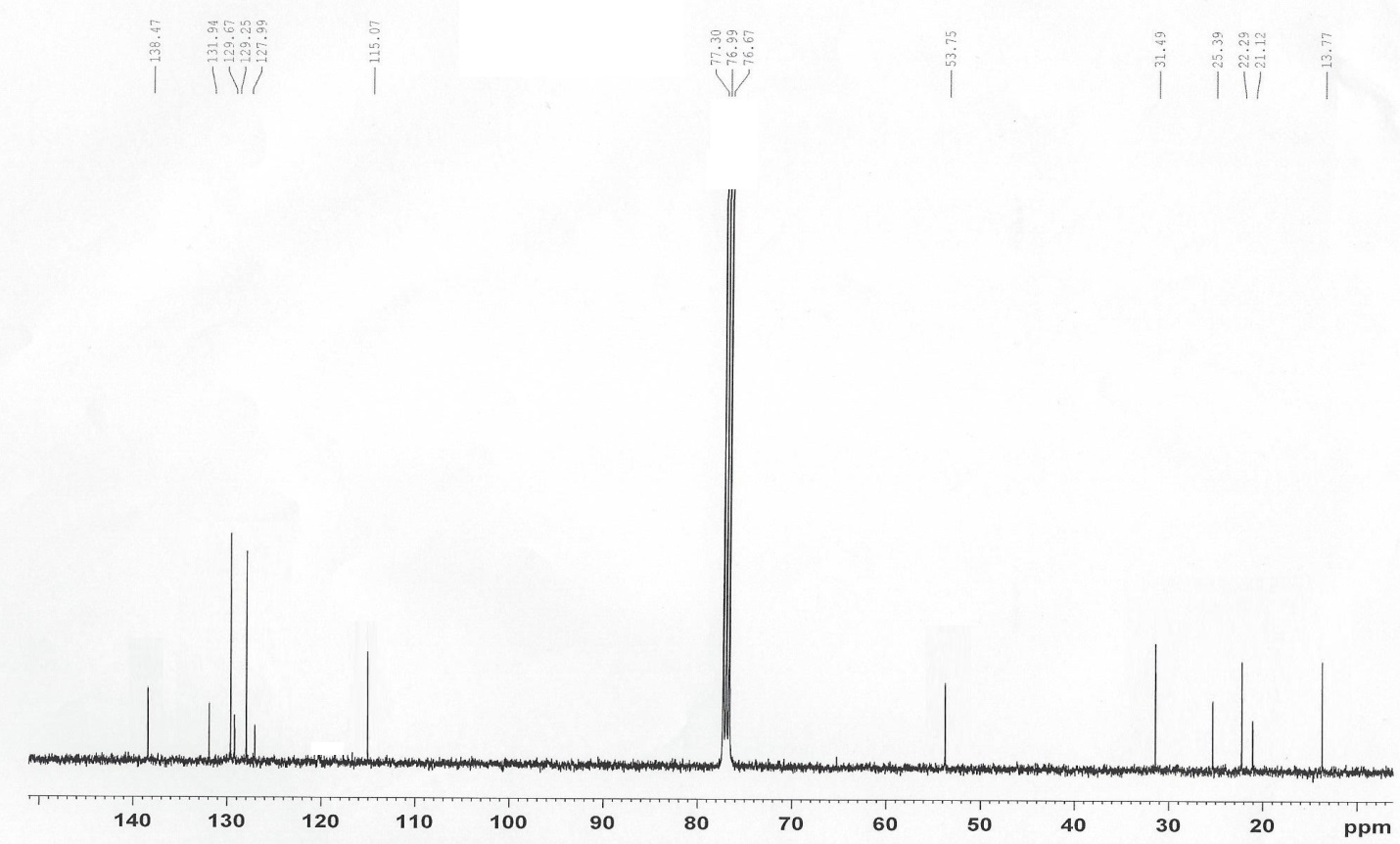


**Figure S4**. ^13^C NMR spectrum of **3ab** in CDCl_3_


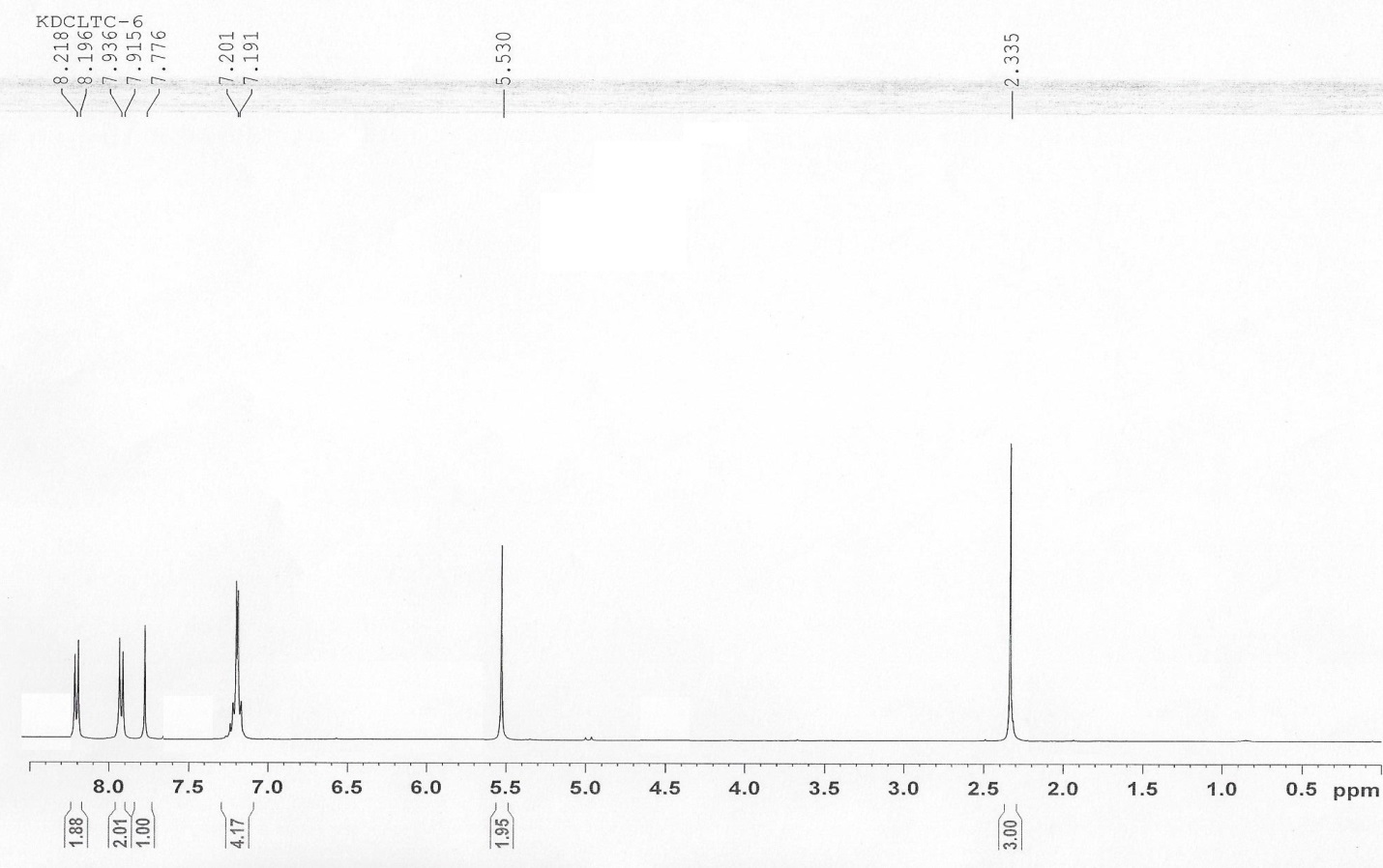


**Figure S5**. ^1^H NMR spectrum of **3ac** in CDCl_3_


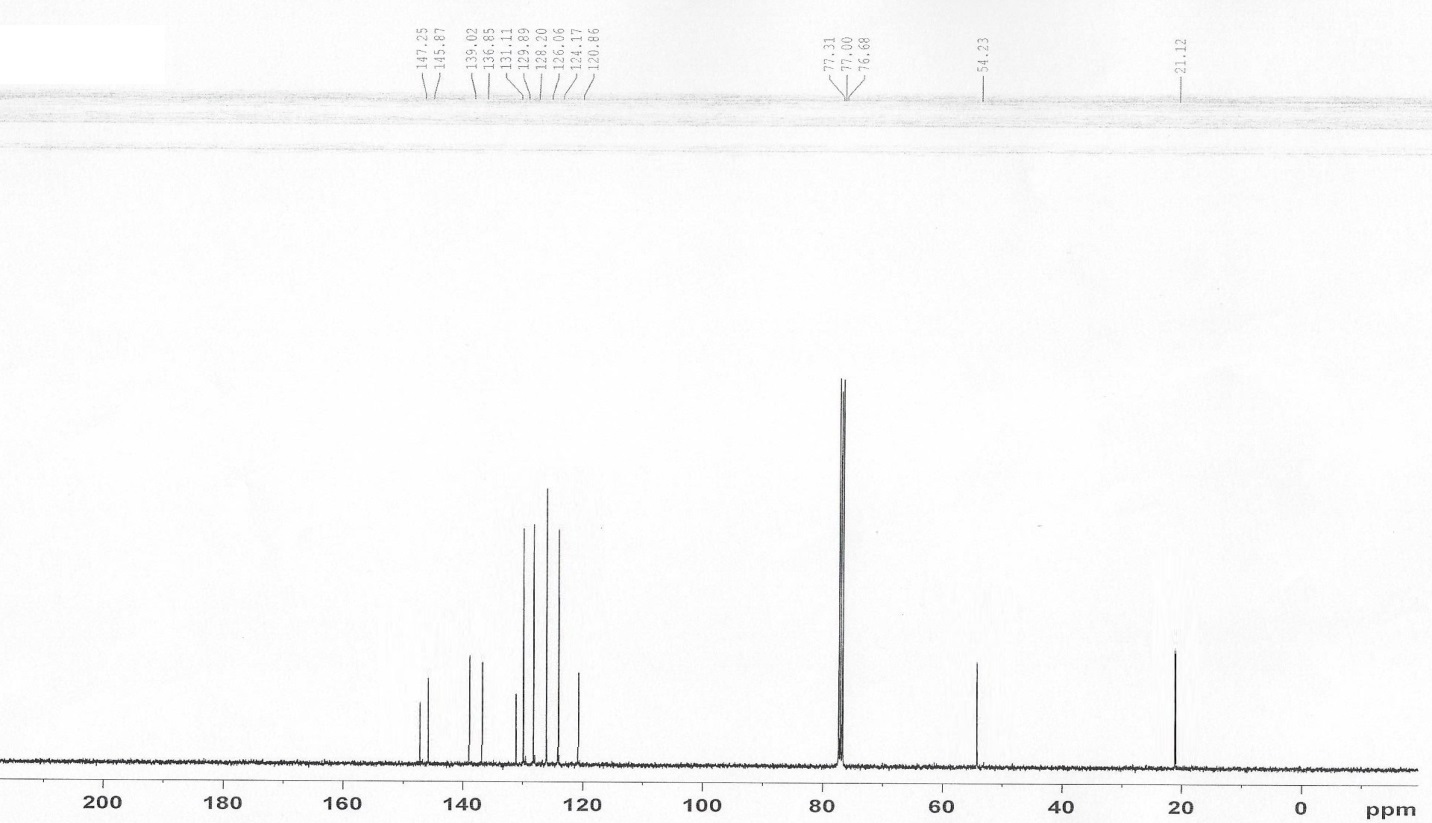


**Figure S6**. ^13^C NMR spectrum of **3ab** in CDCl_3_


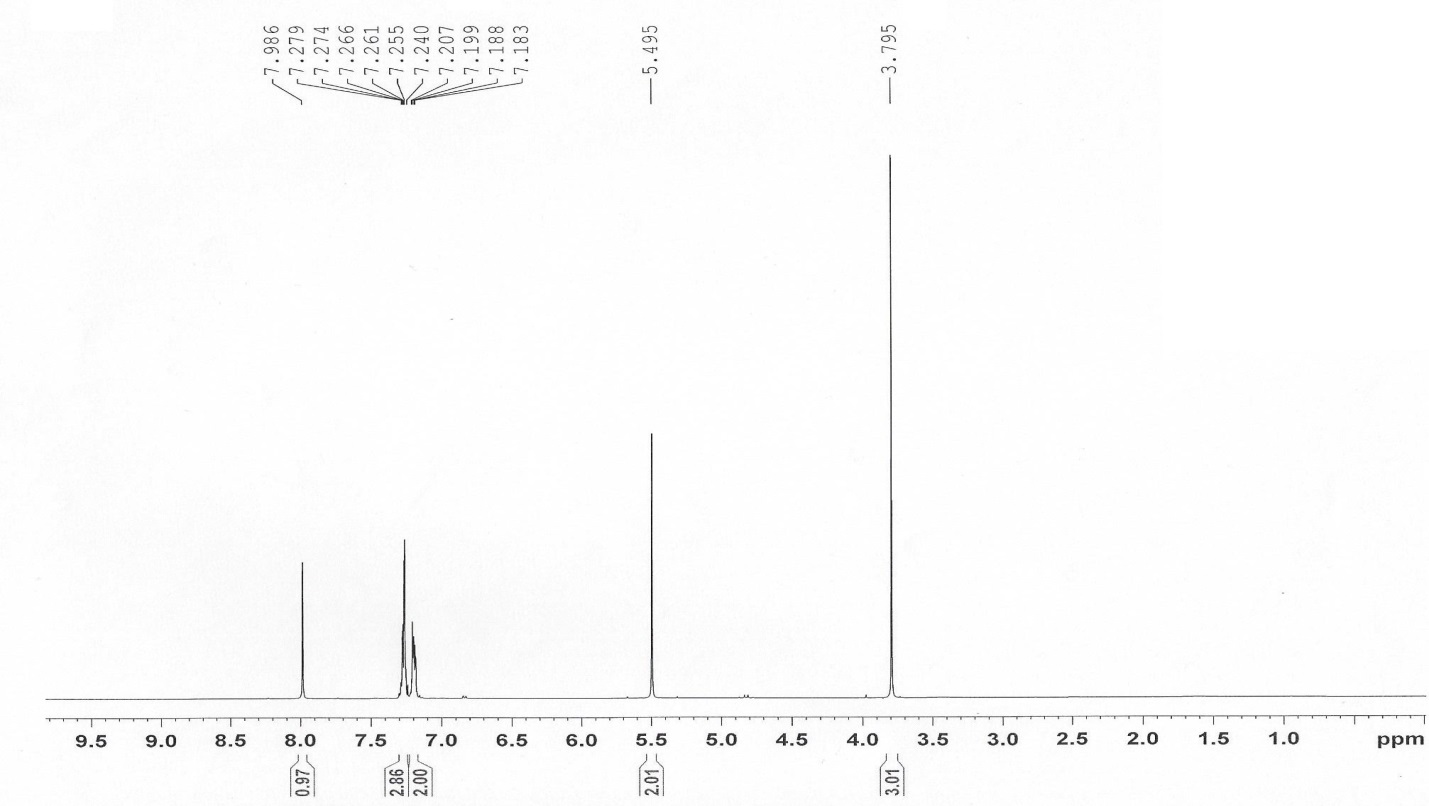


**Figure S7**. ^1^H NMR spectrum of **3bd** in CDCl_3_


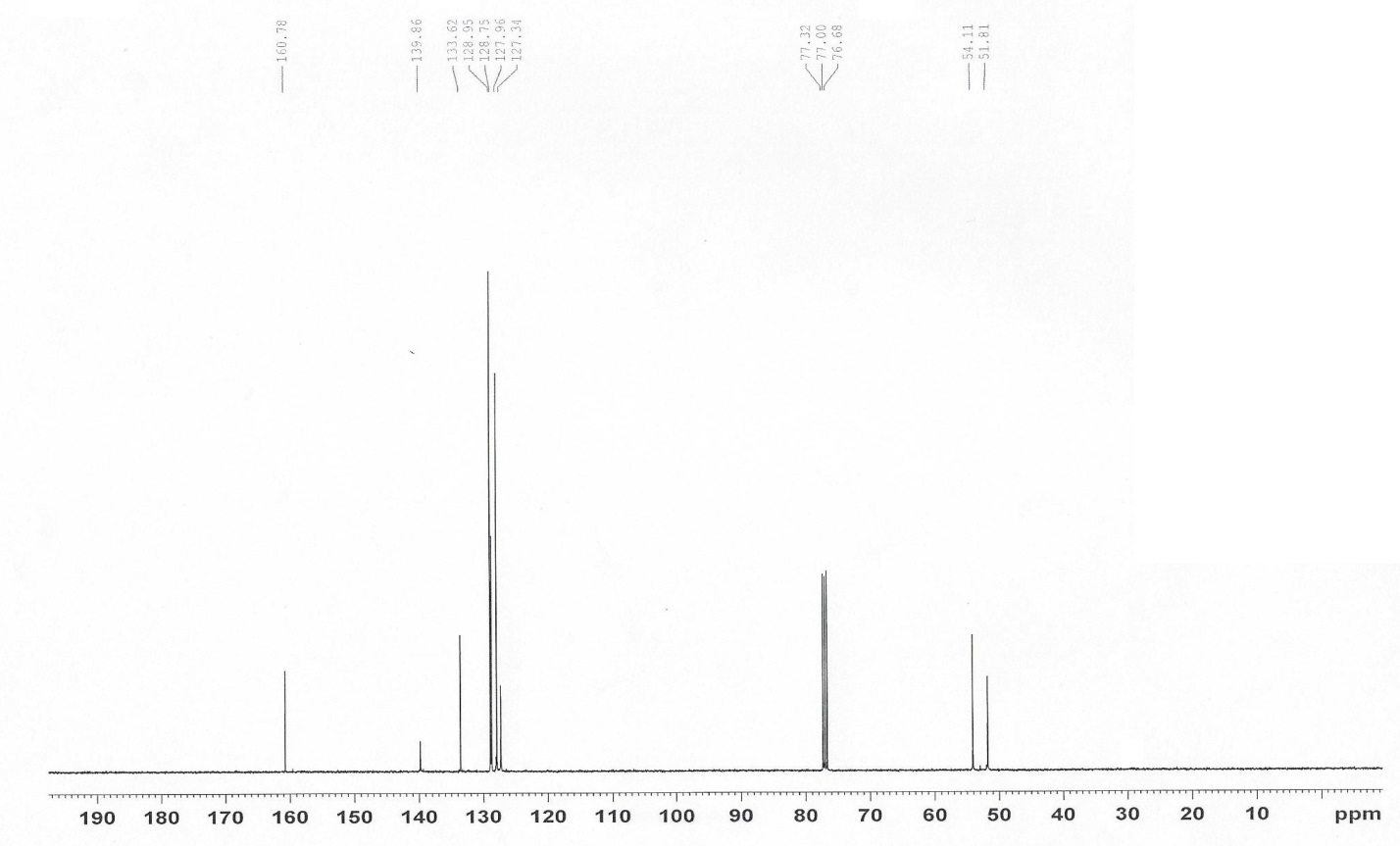


**Figure S8**. ^13^C NMR spectrum of **3bd** in CDCl_3_


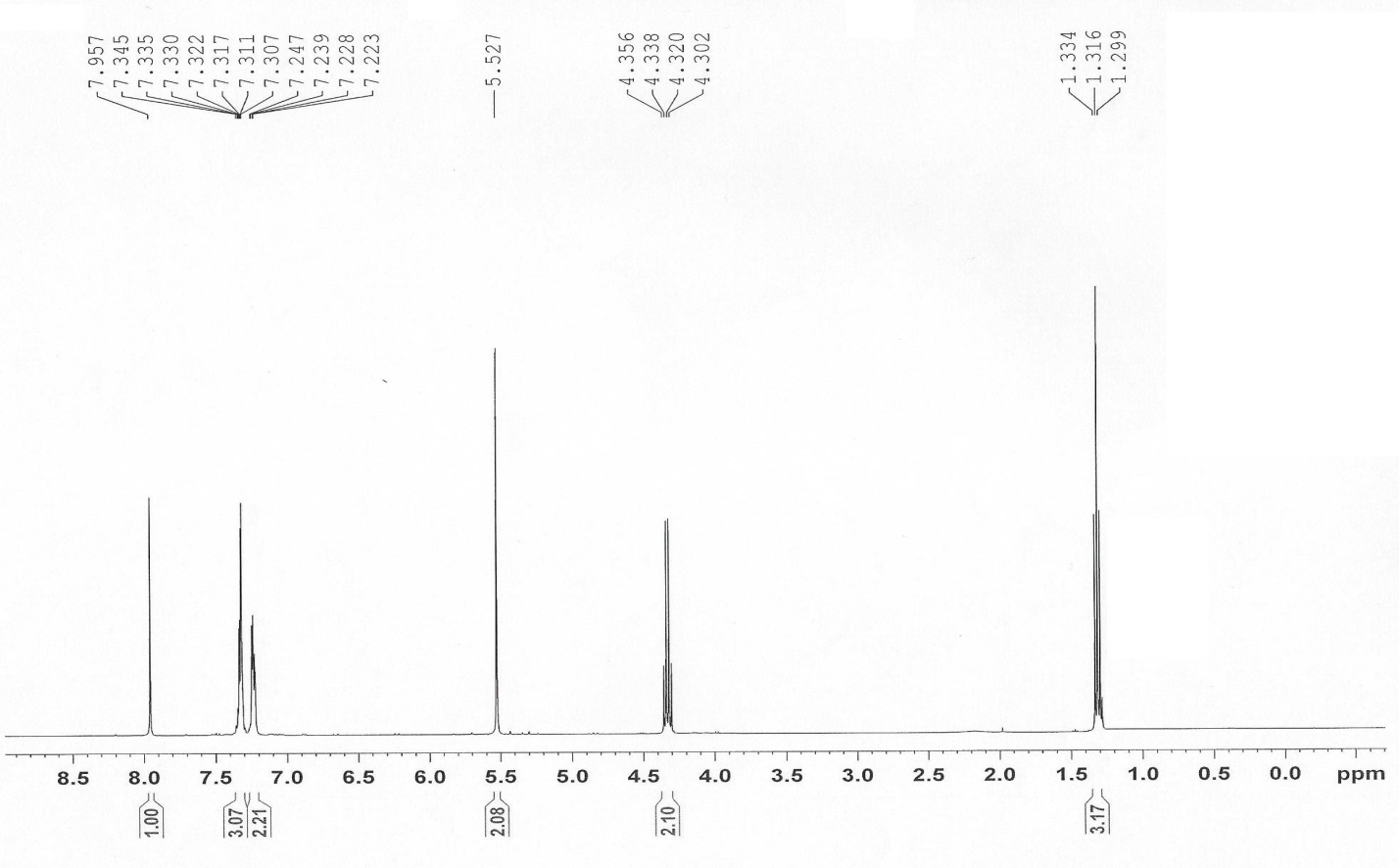


**Figure S9**. ^1^H NMR spectrum of **3be** in CDCl_3_


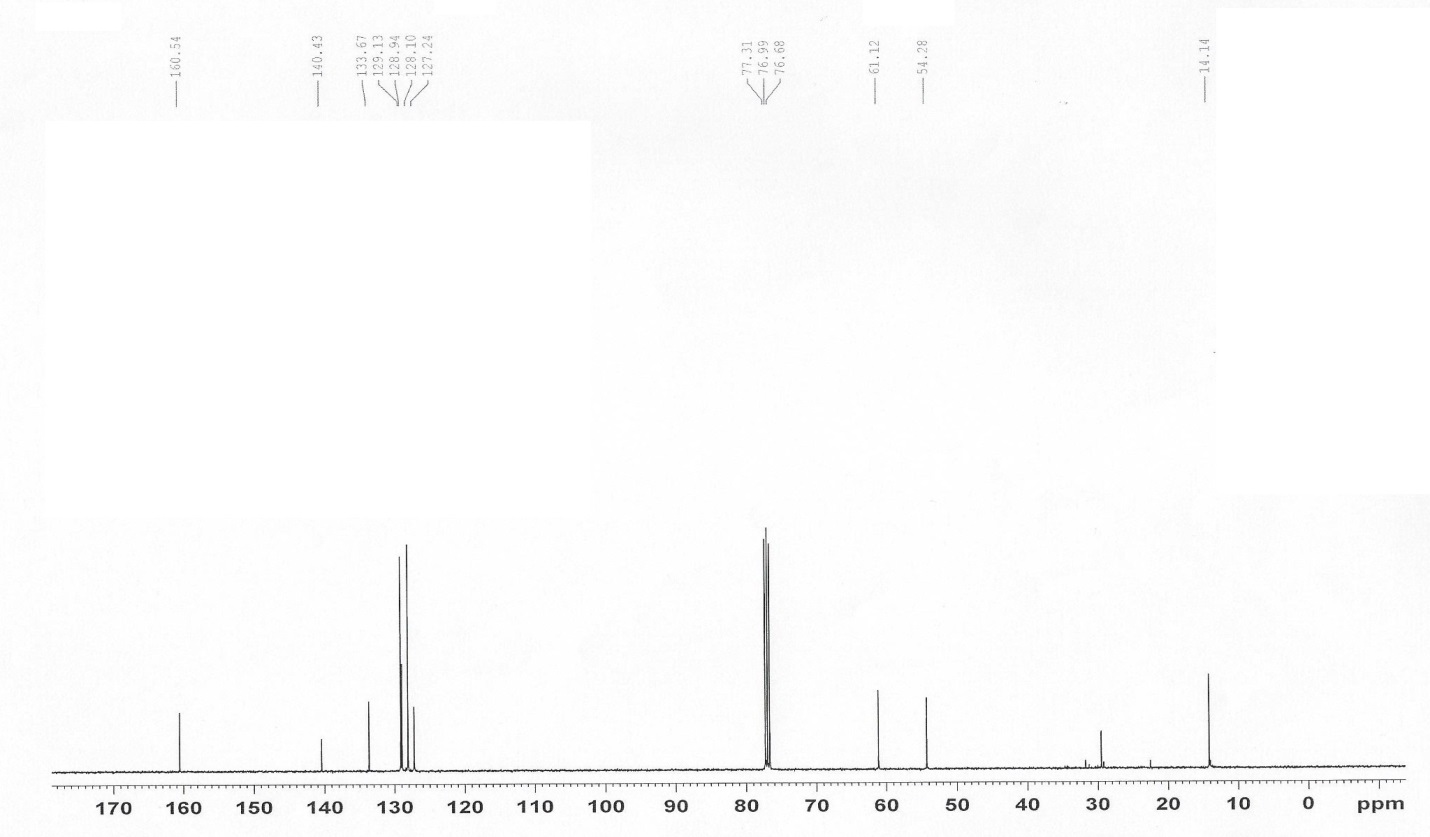


**Figure S10**. ^13^C NMR spectrum of **3be** in CDCl_3_


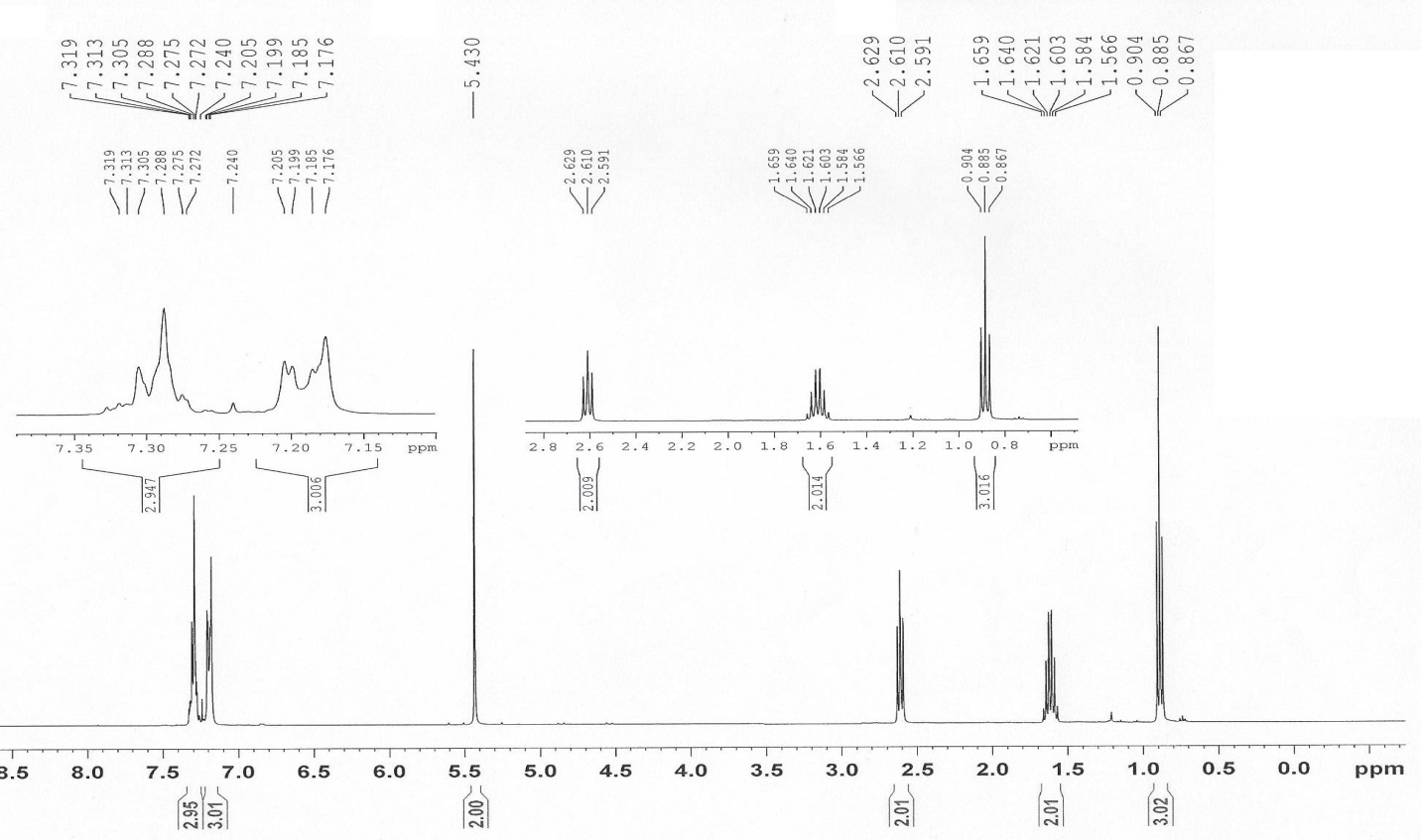


**Figure S11**. ^1^H NMR spectrum of **3bb** in CDCl_3_


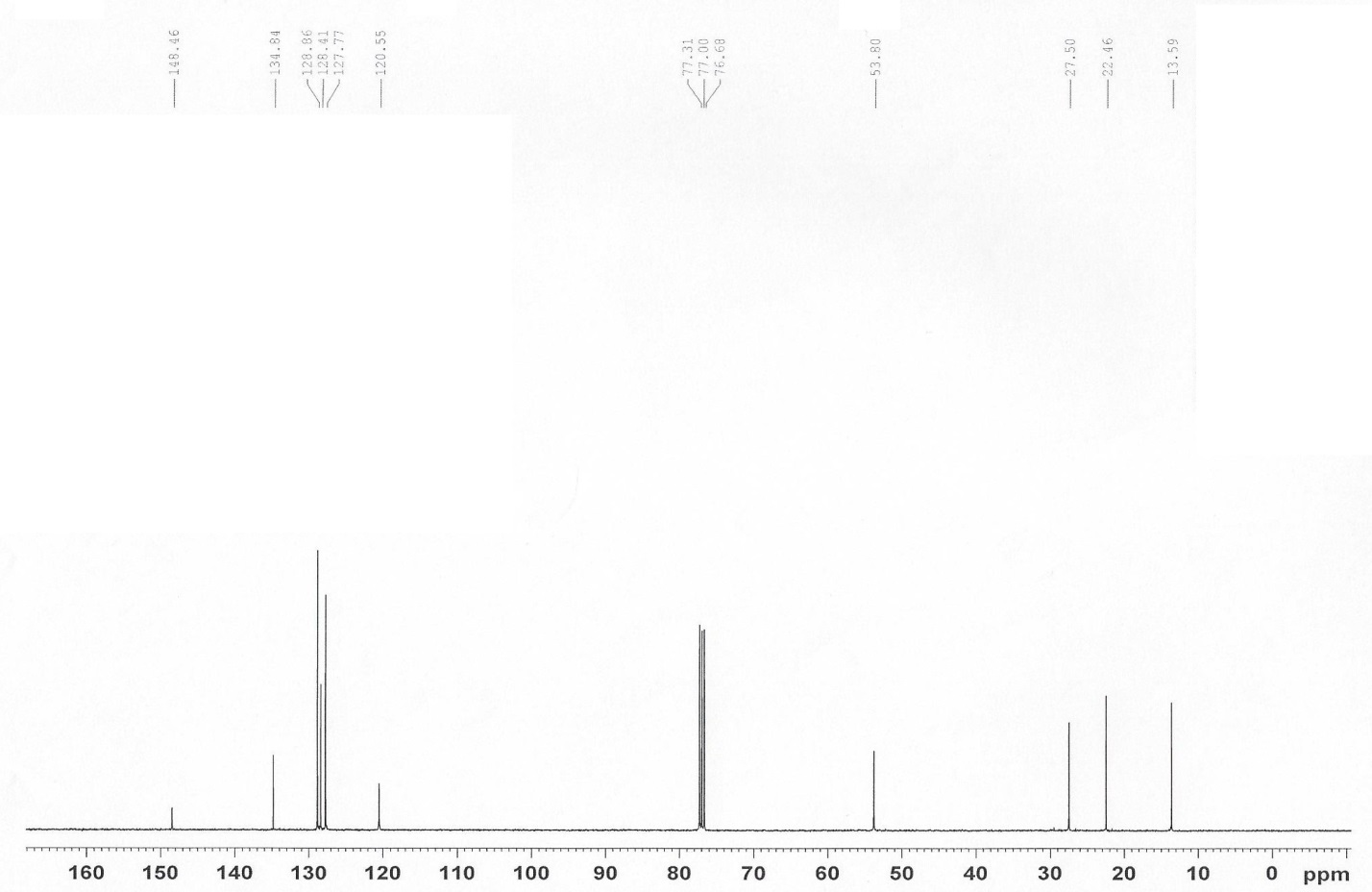


**Figure S12**. ^13^C NMR spectrum of **3bb** in CDCl_3_


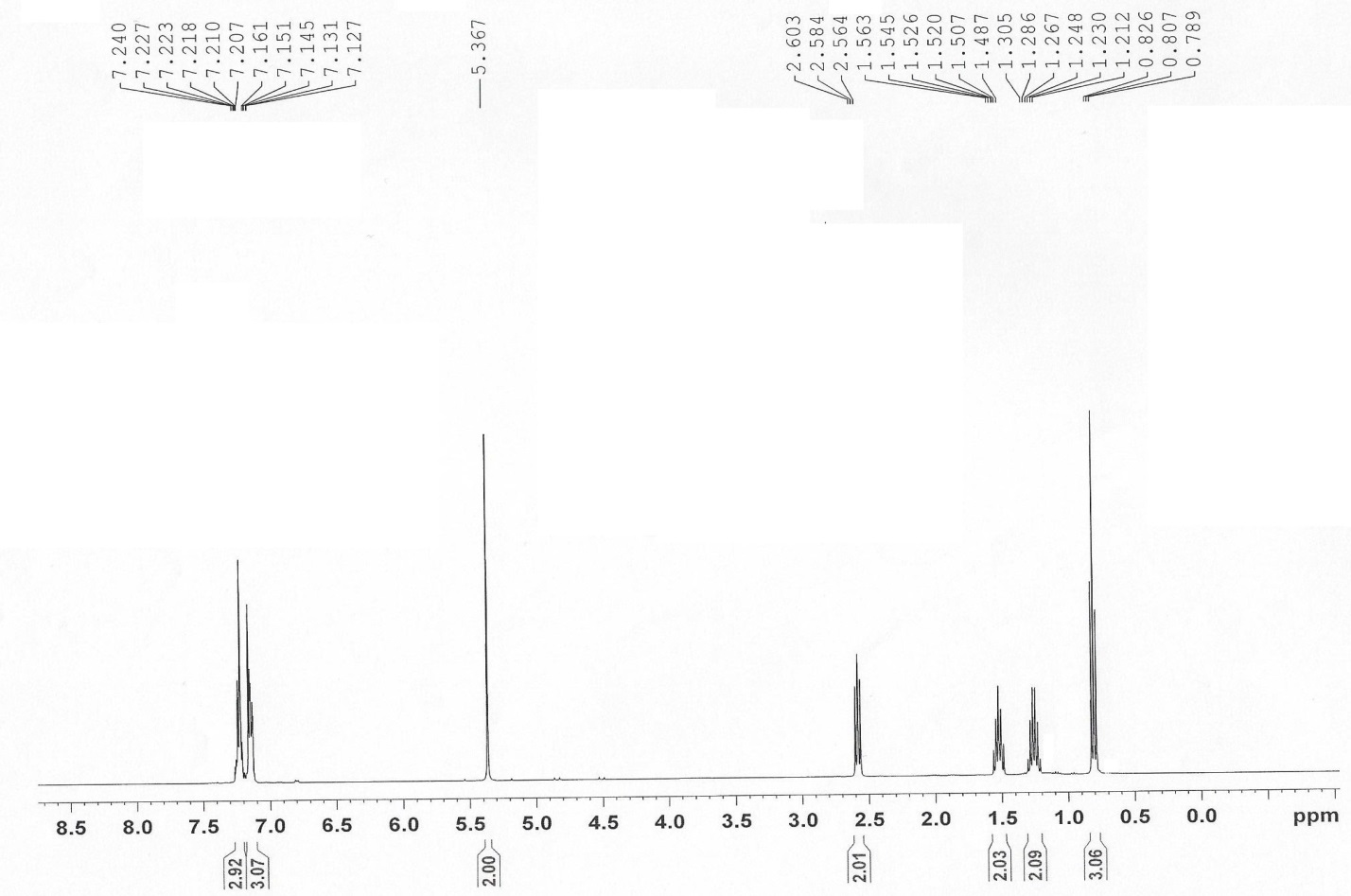


**Figure S13**. ^1^H NMR spectrum of **3bf** in CDCl_3_


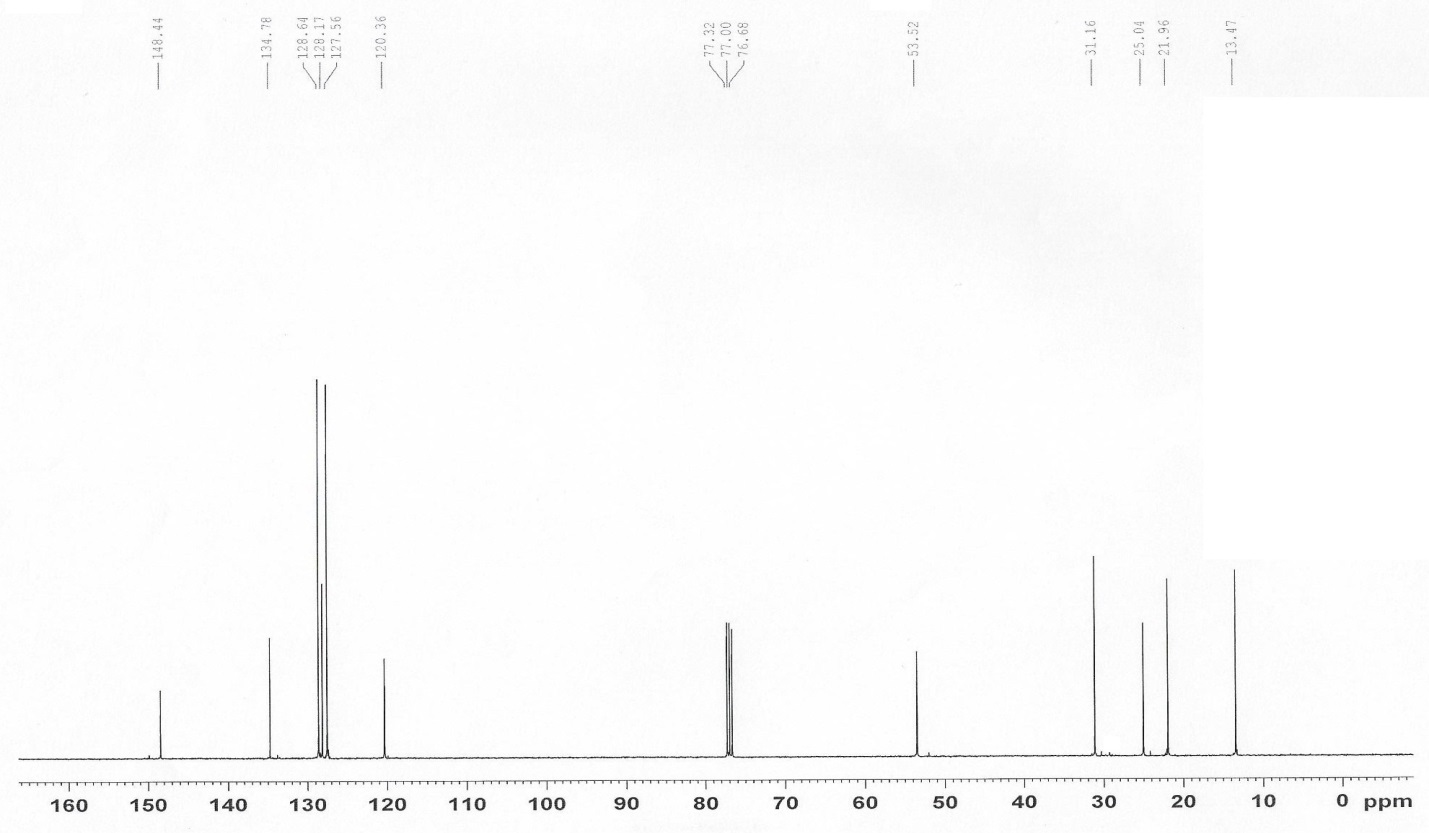


**Figure S14**. ^13^C NMR spectrum of **3bf** in CDCl_3_


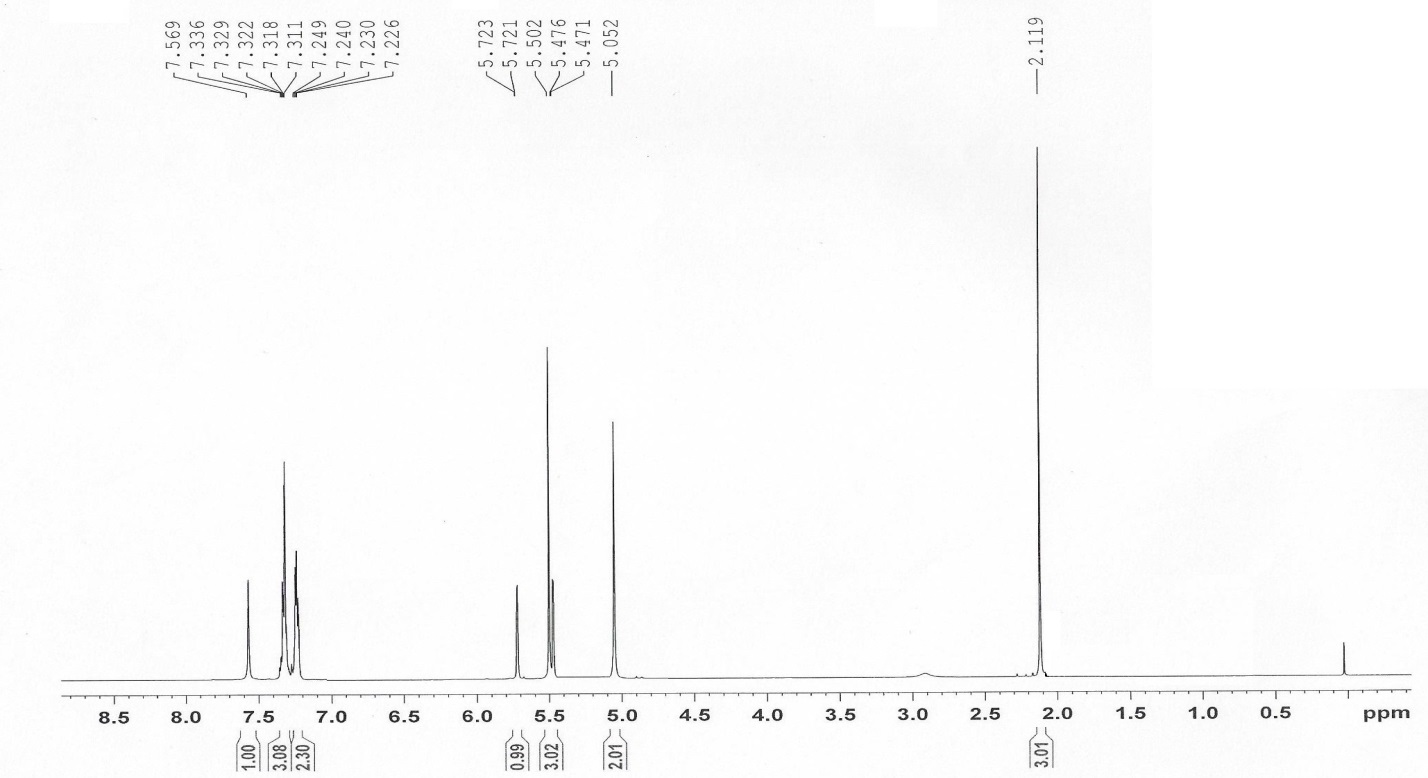


**Figure S15**. ^1^H NMR spectrum of **3bg** in CDCl_3_


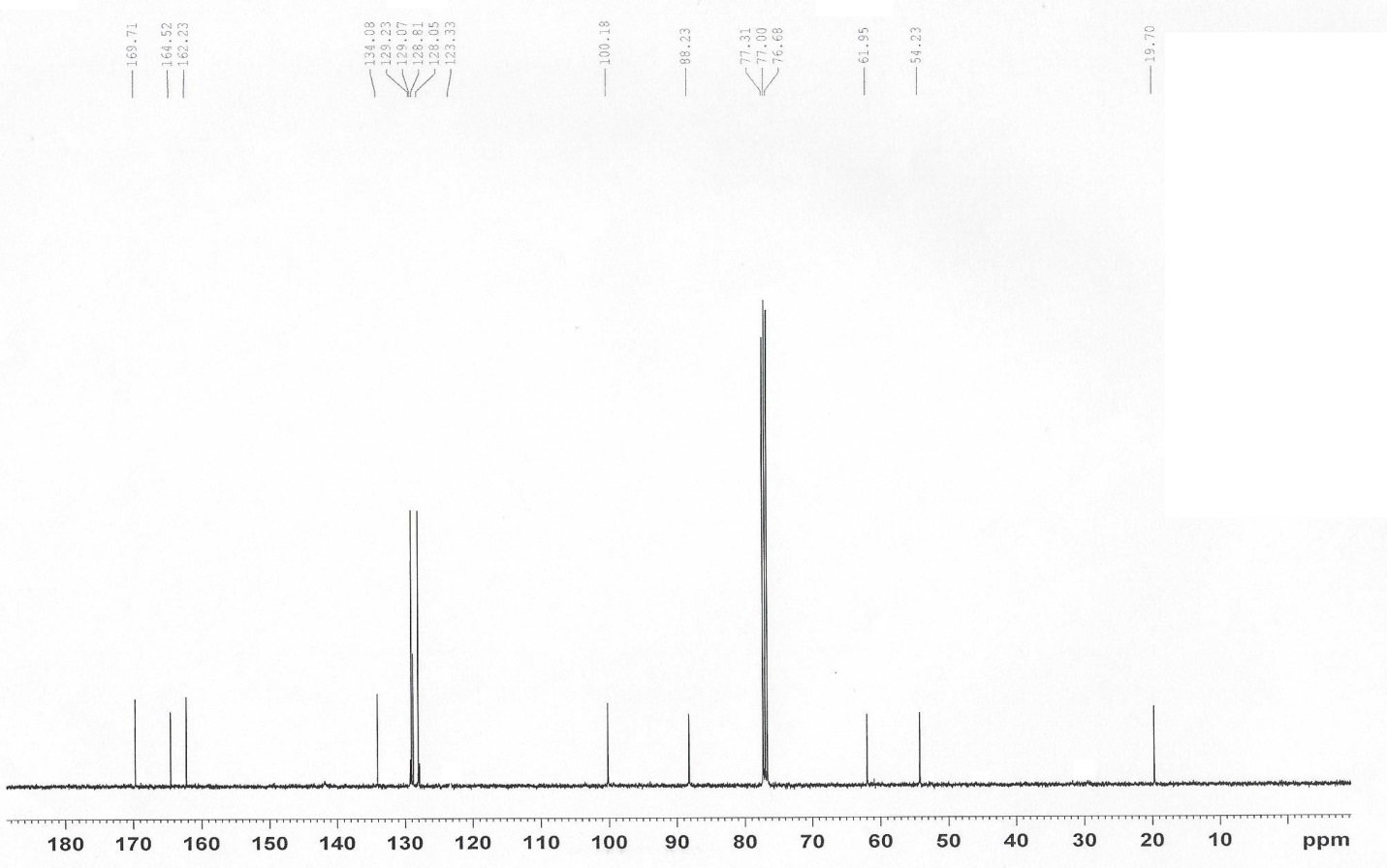


**Figure S16**. ^13^C NMR spectrum of **3bg** in CDCl_3_


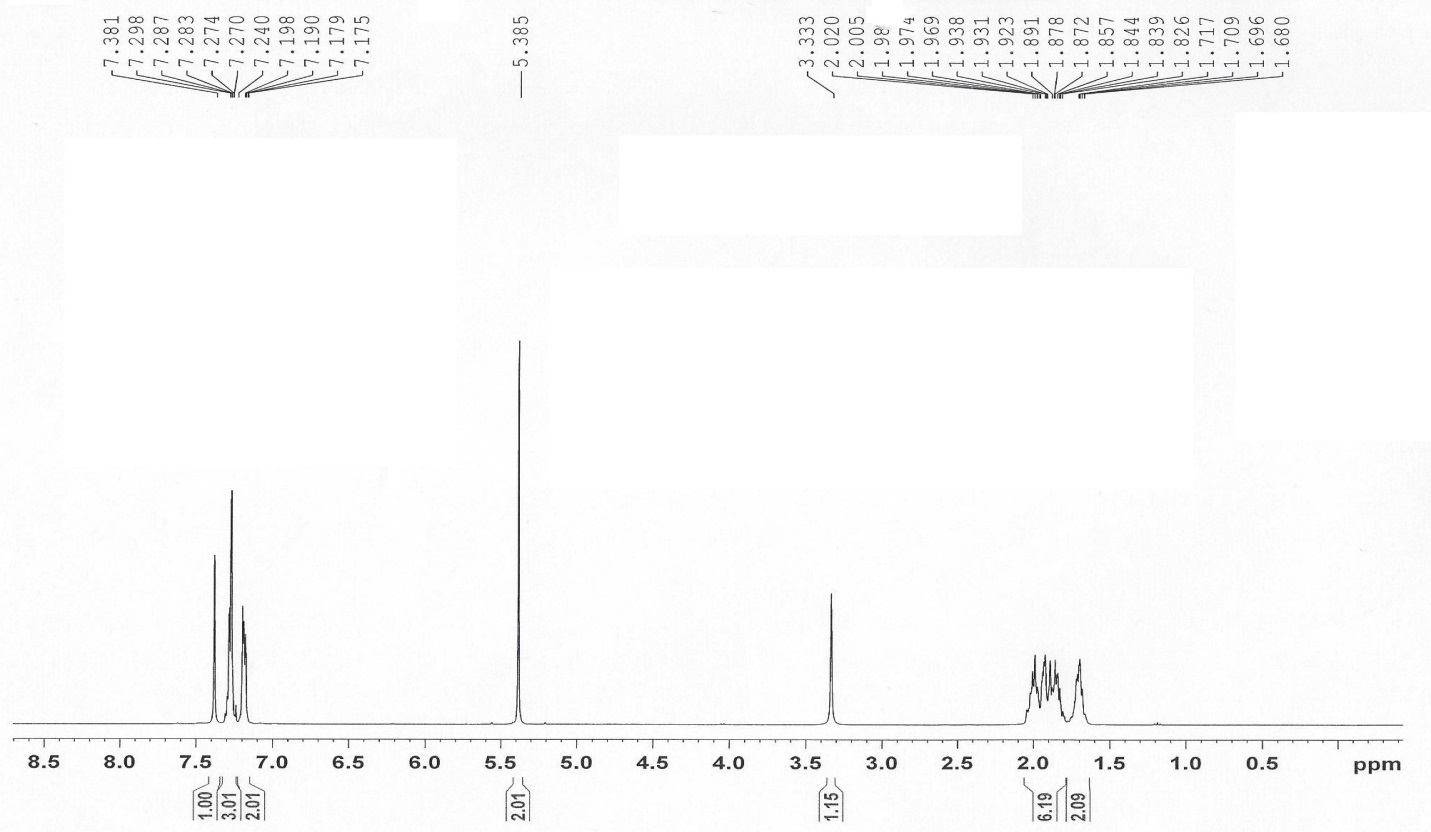


**Figure S17**. ^1^H NMR spectrum of **3bh** in CDCl_3_


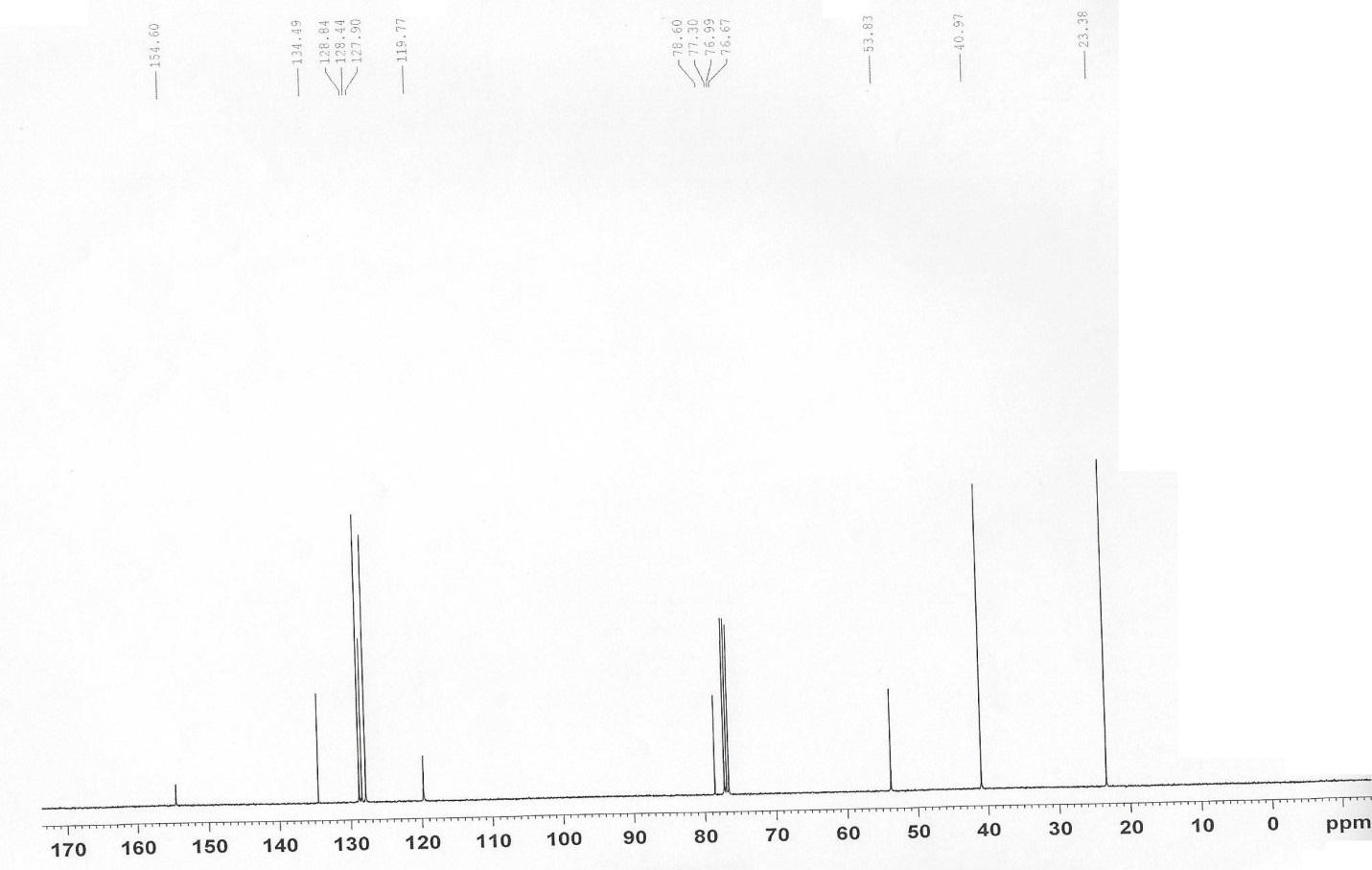


**Figure S18**. ^13^C NMR spectrum of **3bh** in CDCl_3_


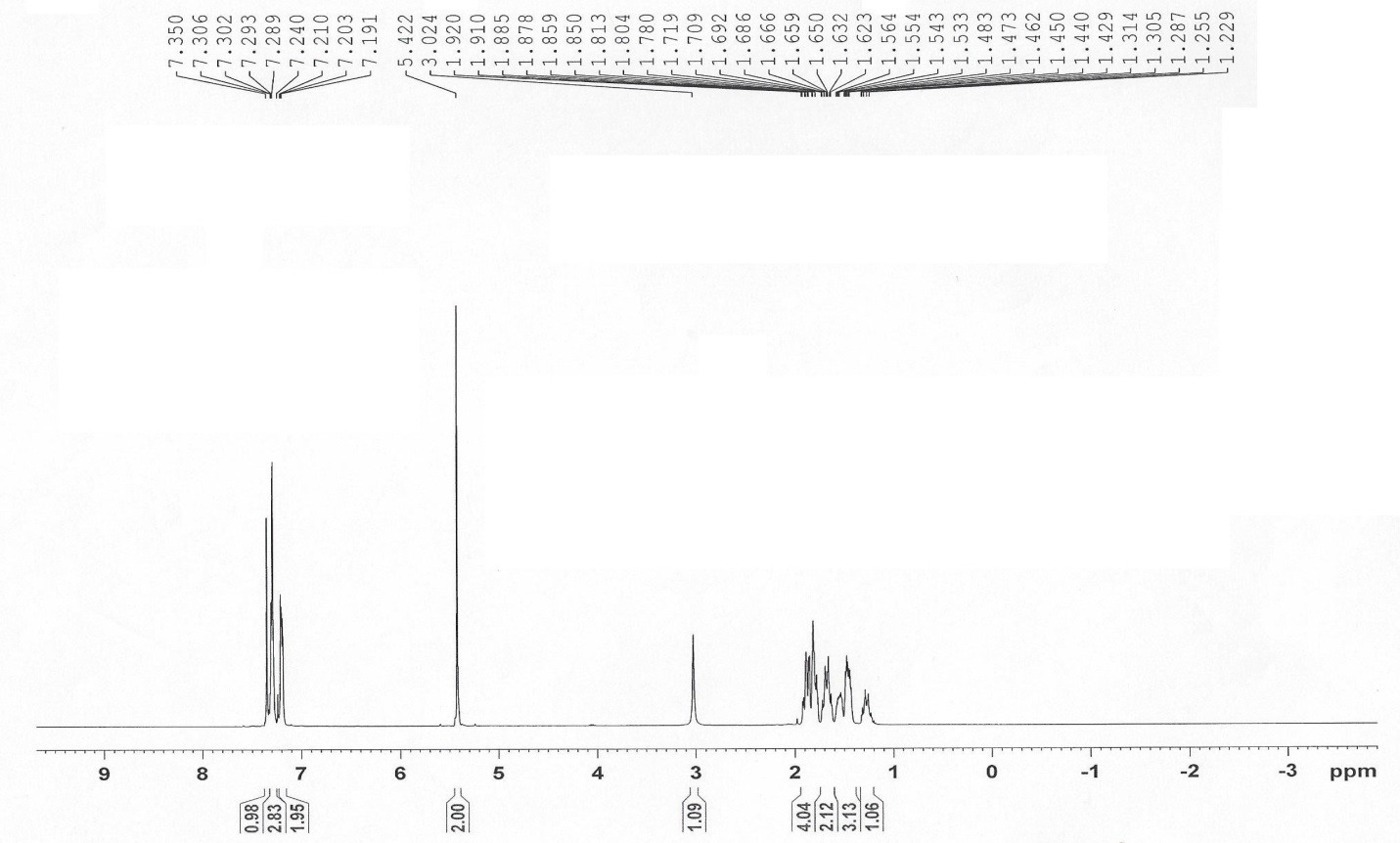


**Figure S19**. ^1^H NMR spectrum of **3bi** in CDCl_3_


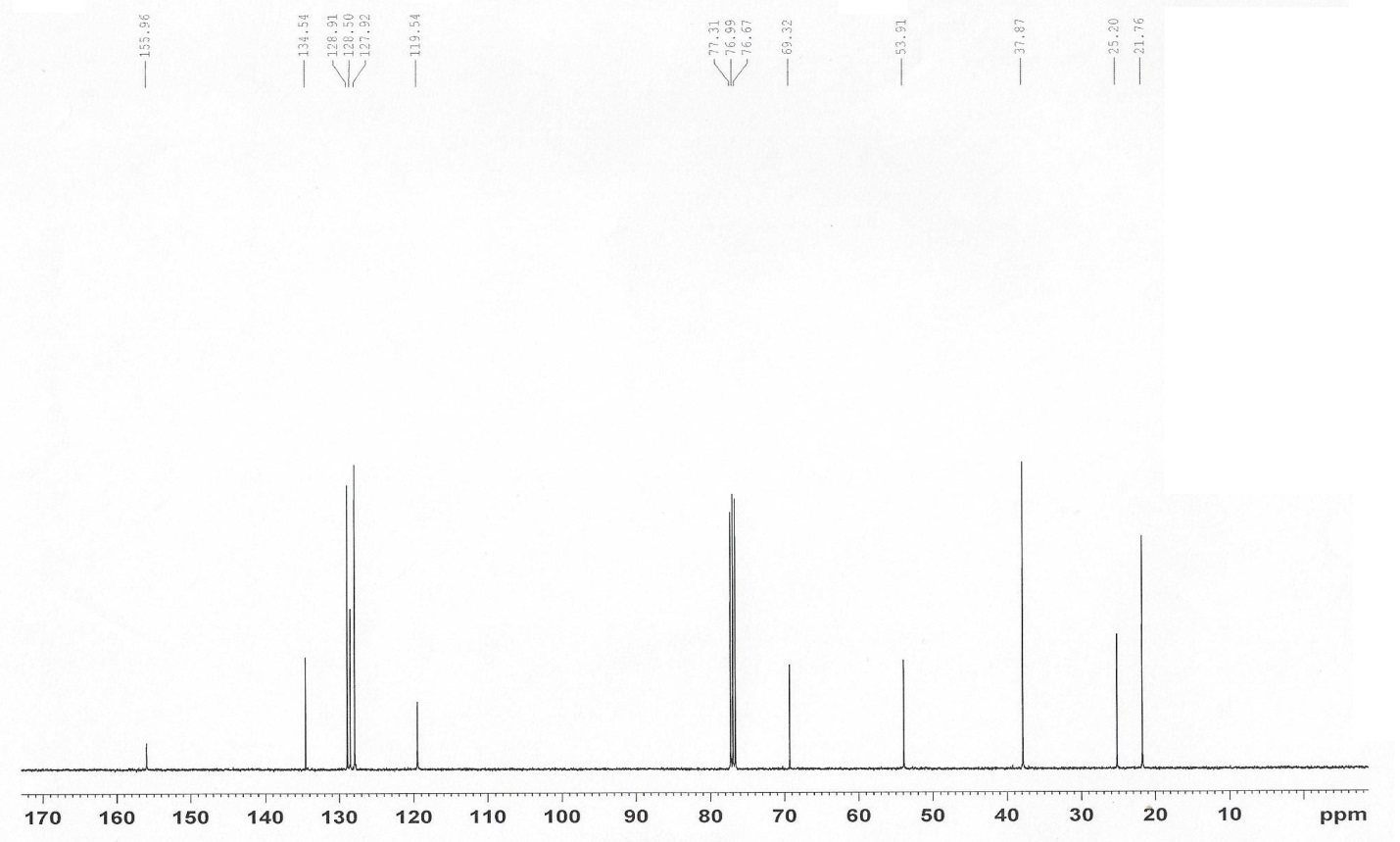


**Figure S20**. ^13^C NMR spectrum of **3bi** in CDCl_3_


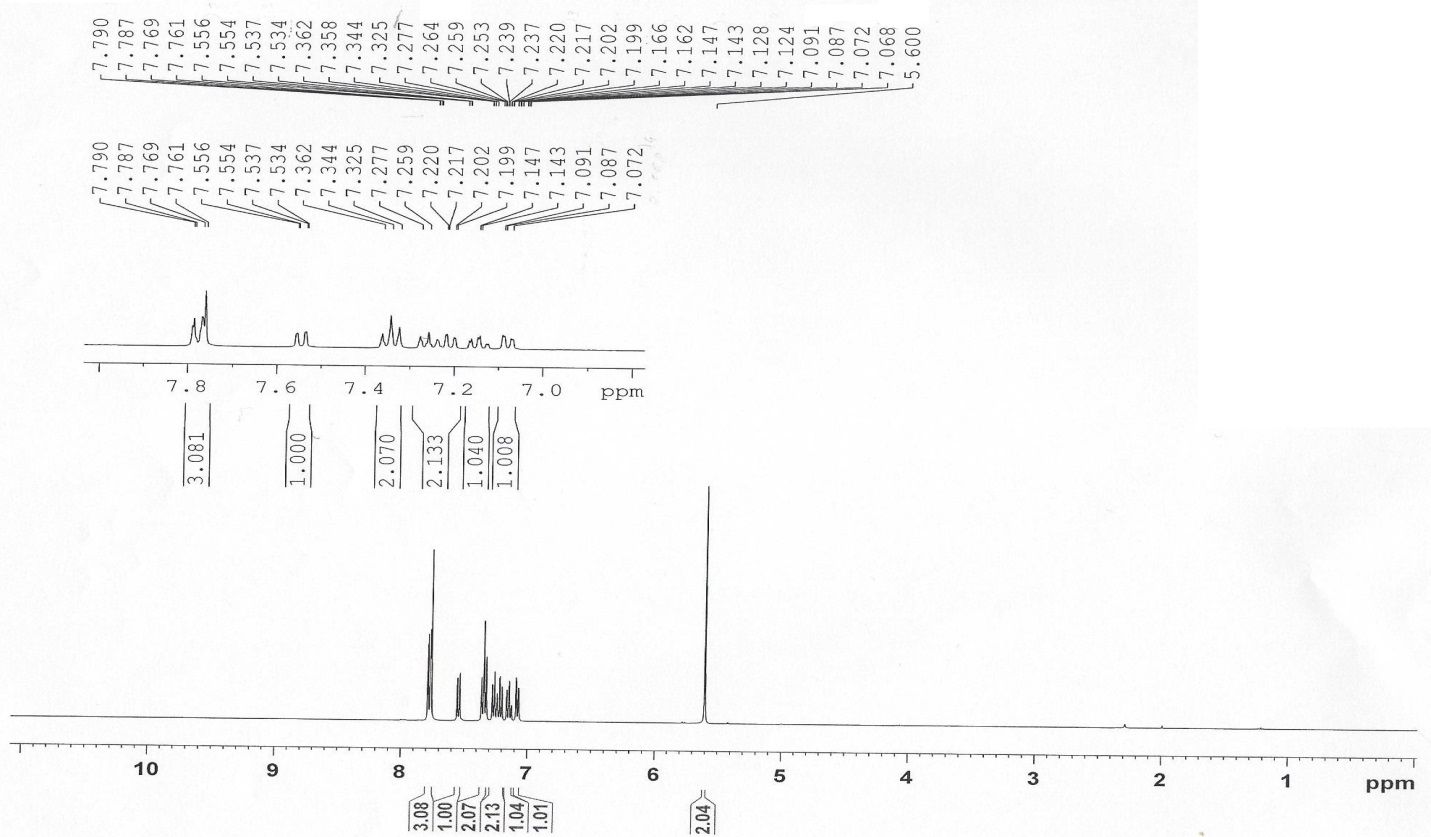


**Figure S21**. ^1^H NMR spectrum of **3cj** in CDCl_3_


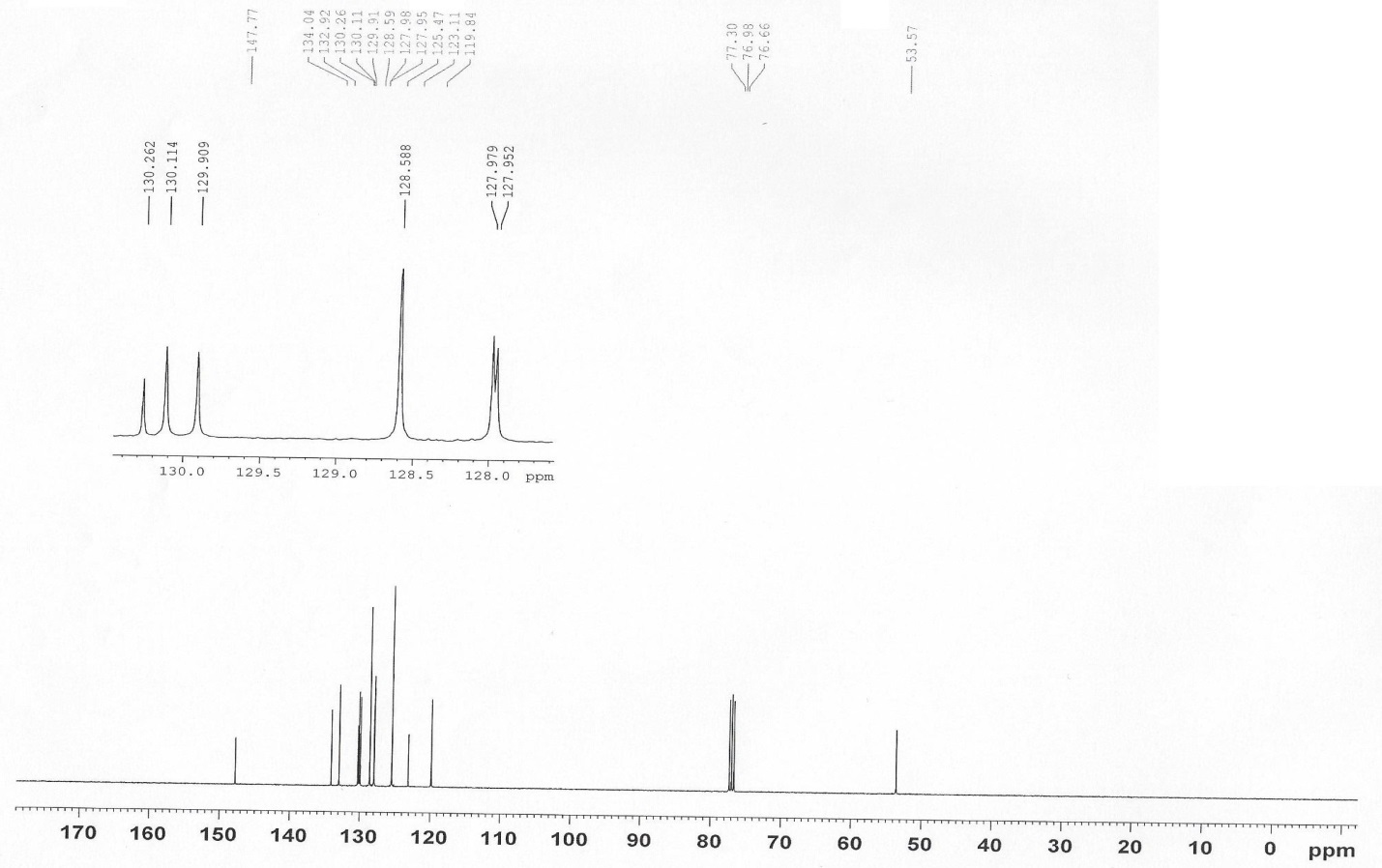


**Figure S22**. ^13^C NMR spectrum of **3bj** in CDCl_3_


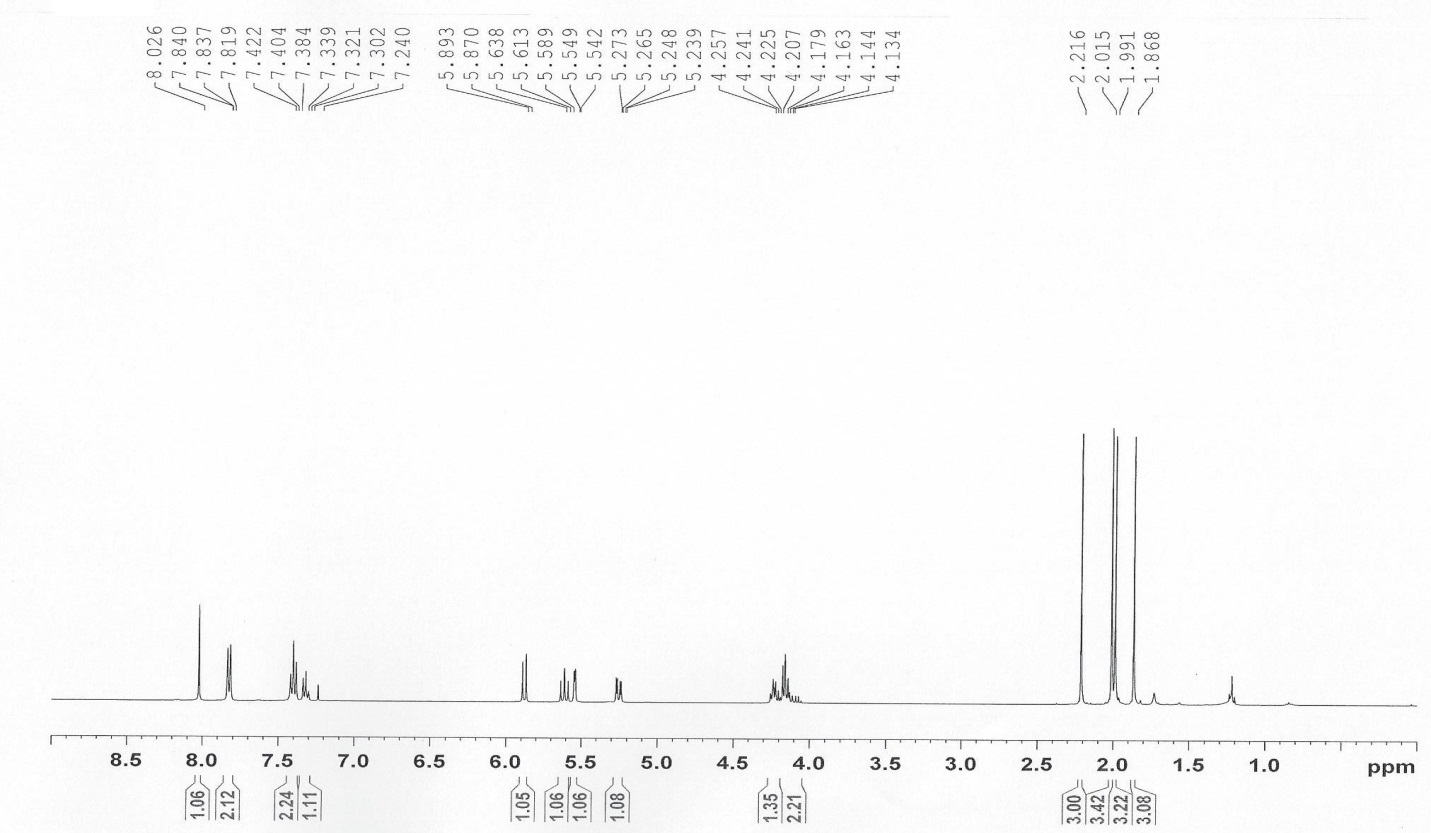


**Figure S23**. ^1^H NMR spectrum of **3dj** in CDCl_3_


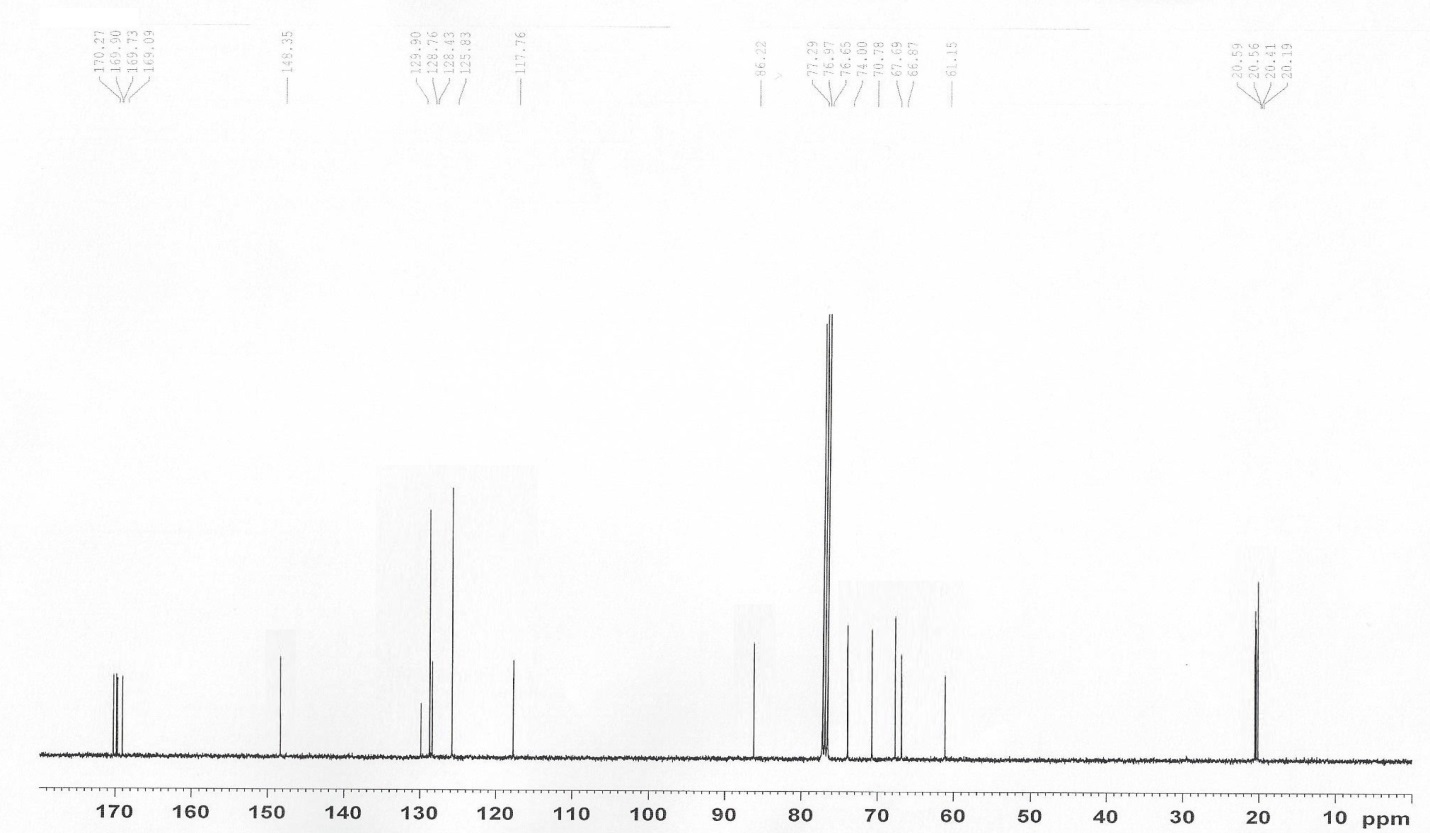


**Figure S24**. ^13^C NMR spectrum of **3dj** in CDCl_3_


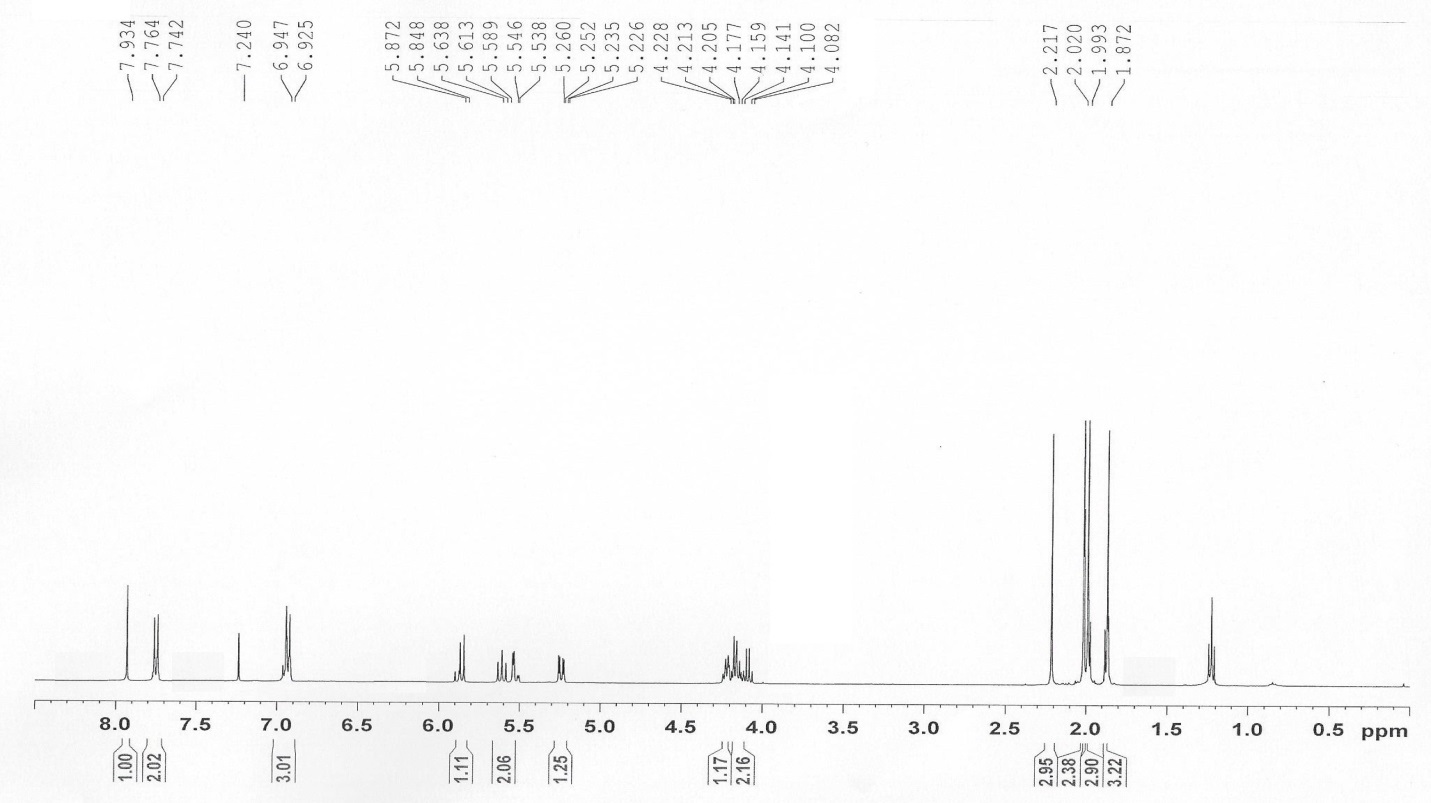


**Figure S25**. ^1^H NMR spectrum of **3ej** in CDCl_3_


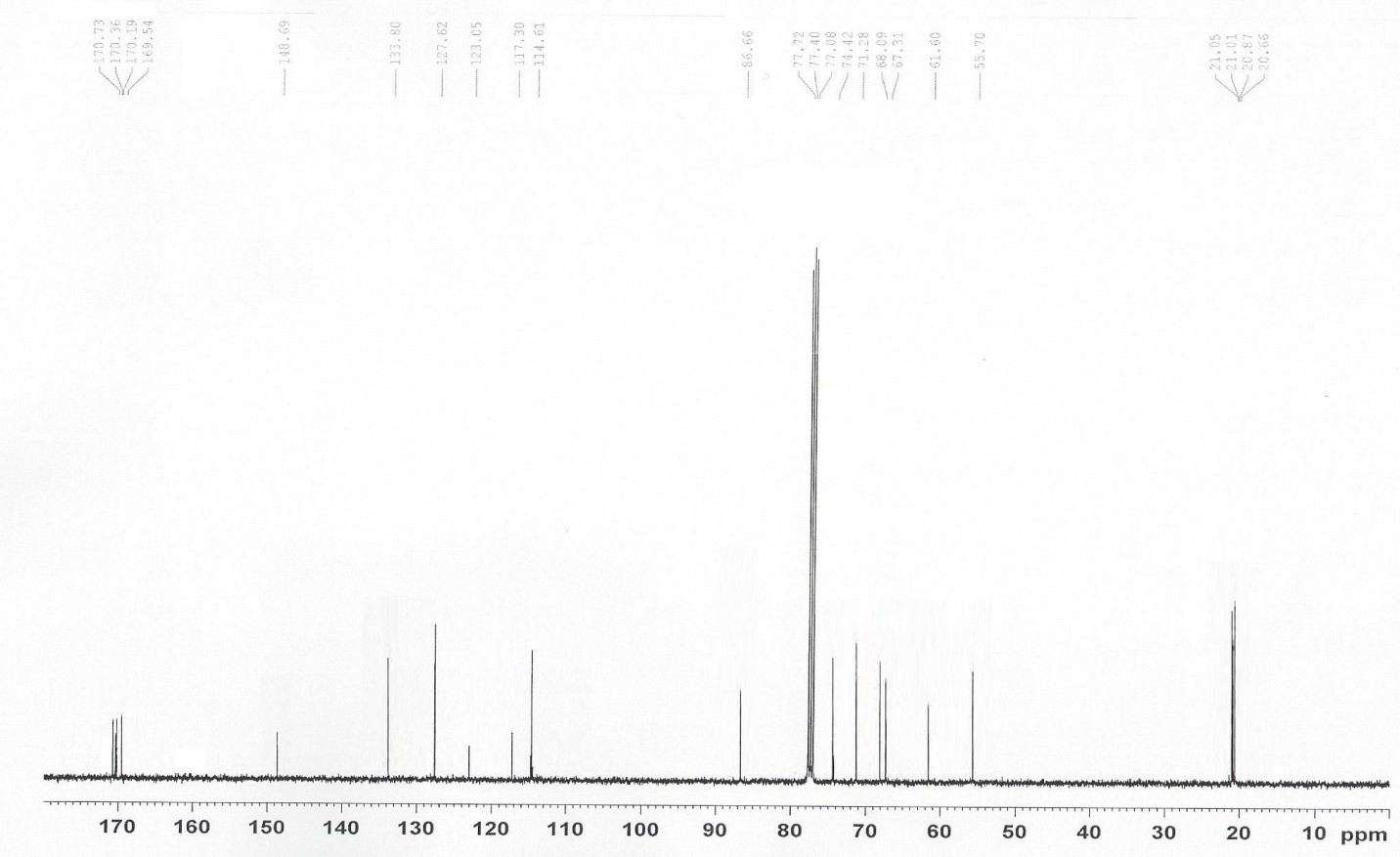


**Figure S26**. ^13^C NMR spectrum of **3dj** in CDCl_3_

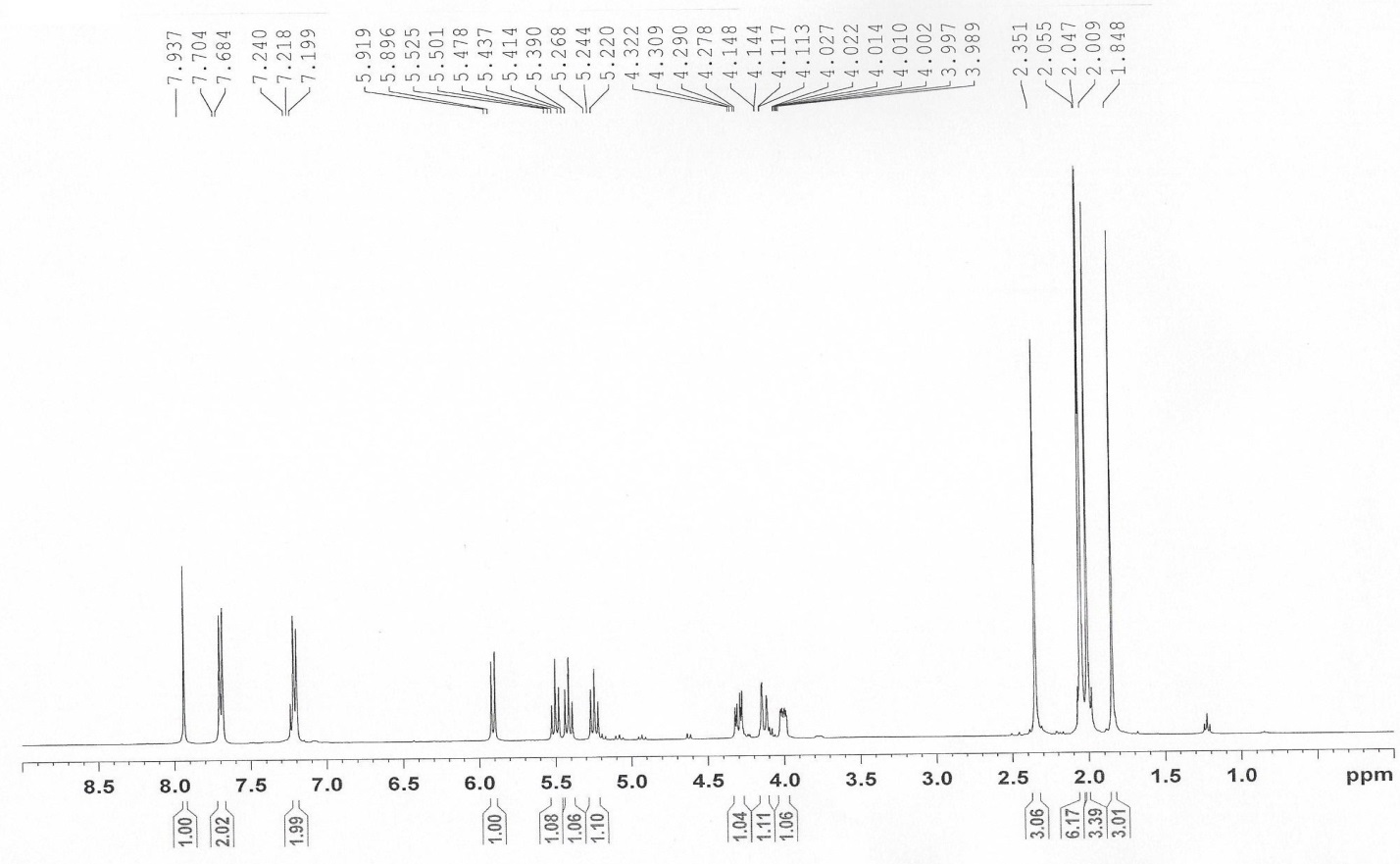


**Figure S27**. ^1^H NMR spectrum of **3da** in CDCl_3_


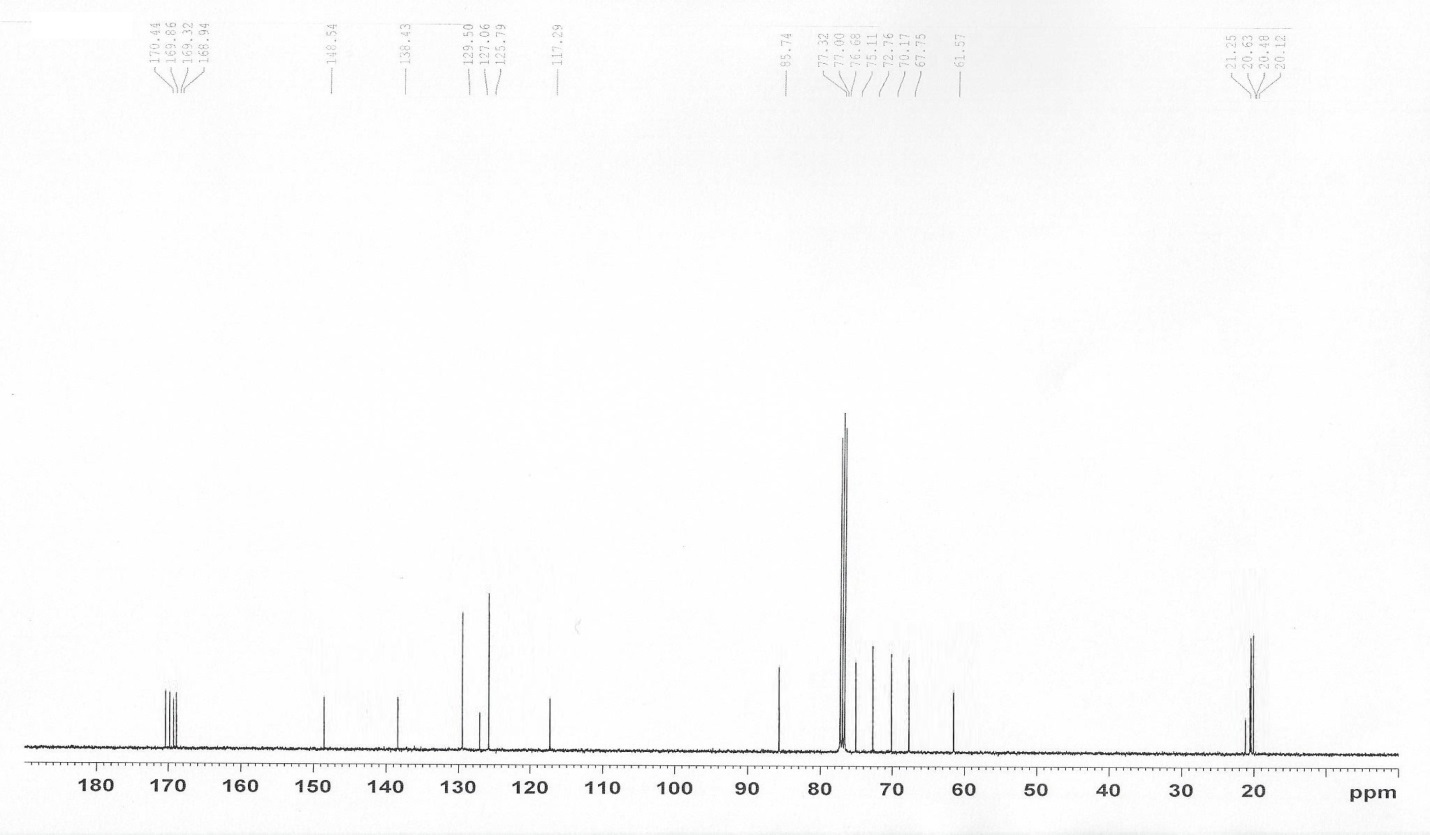


**Figure S28**. ^13^C NMR spectrum of **3da** in CDCl_3_


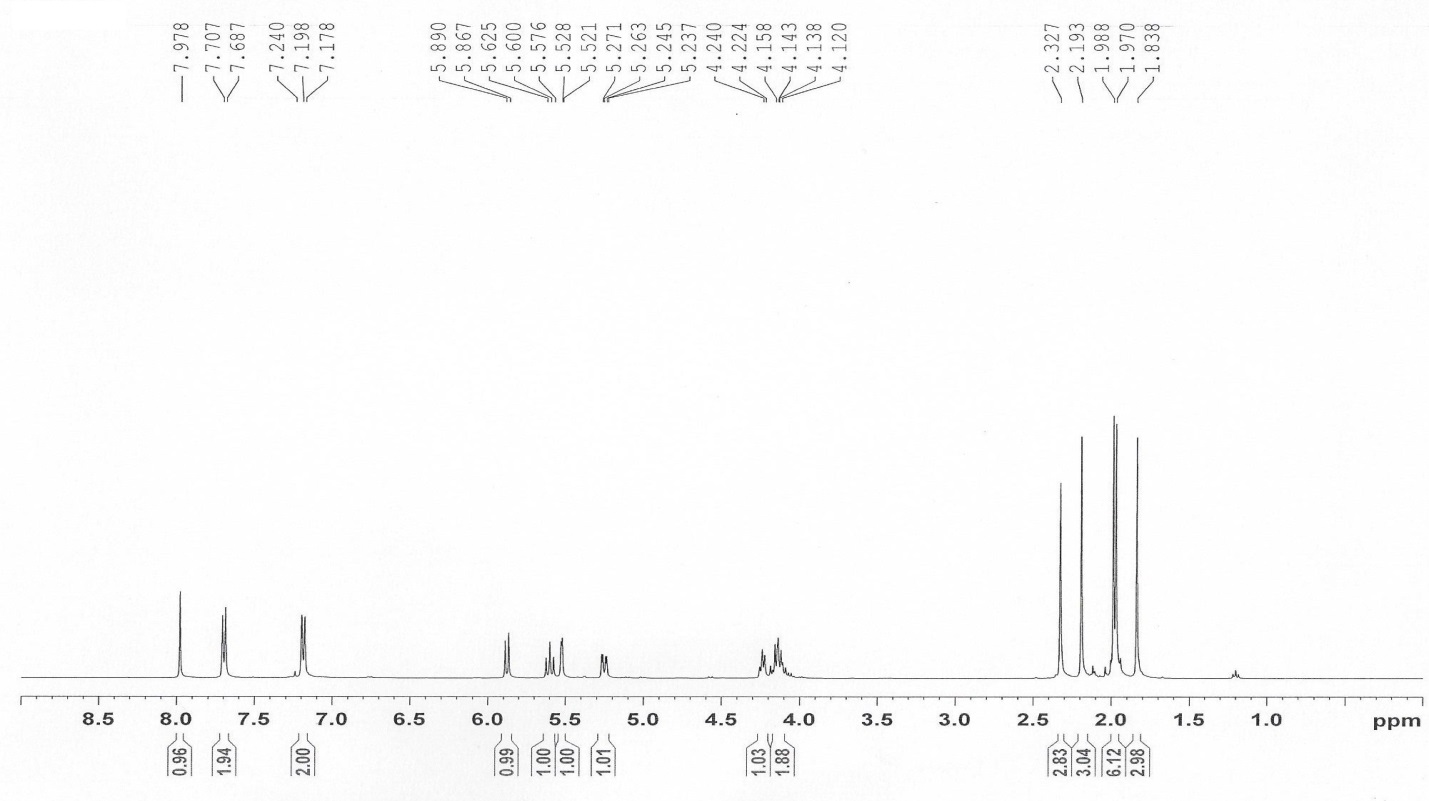


**Figure S29**. ^1^H NMR spectrum of **3ea** in CDCl_3_


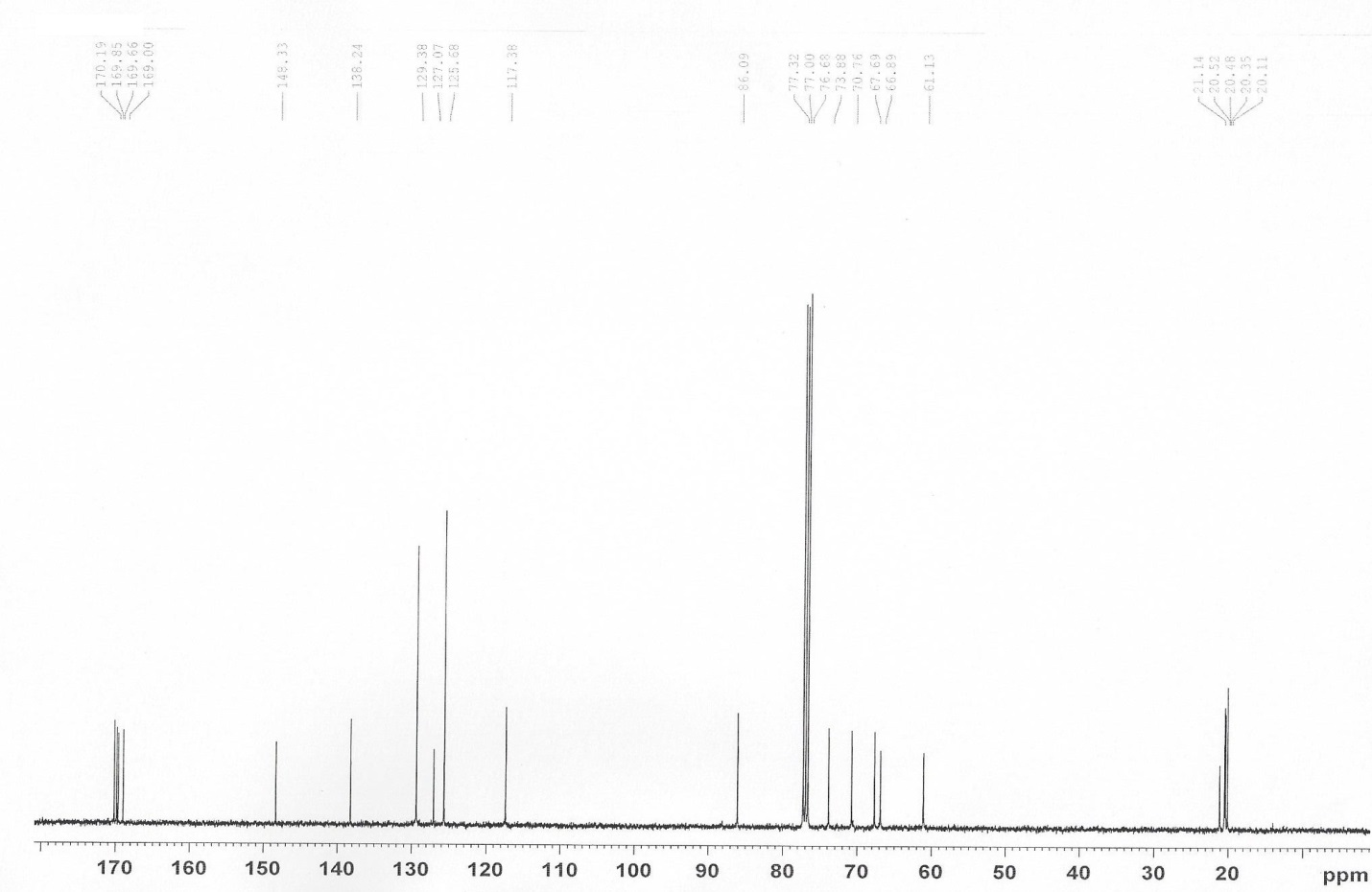


**Figure S30**. ^13^C NMR spectrum of **3ea** in CDCl_3_


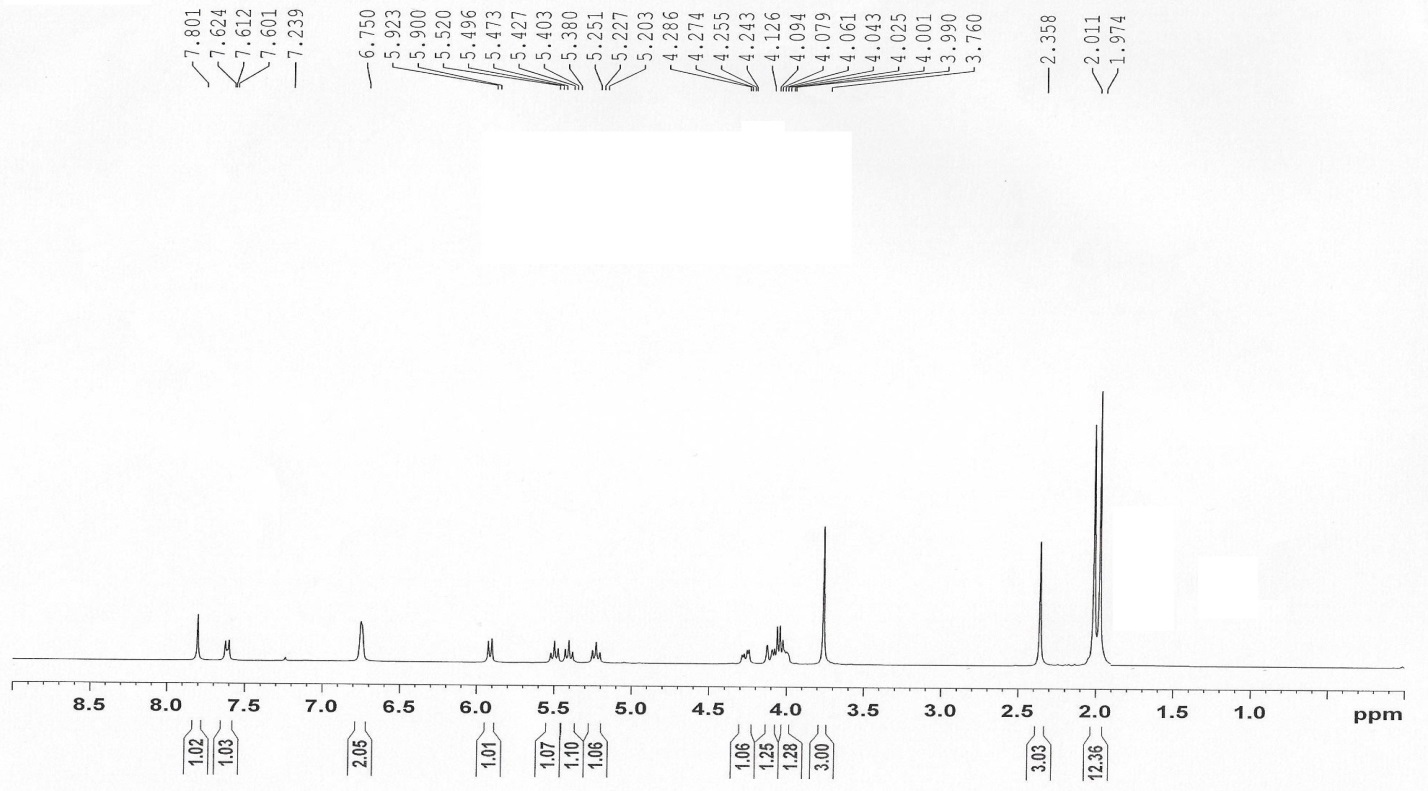


**Figure S31**. ^1^H NMR spectrum of **3dk** in CDCl_3_


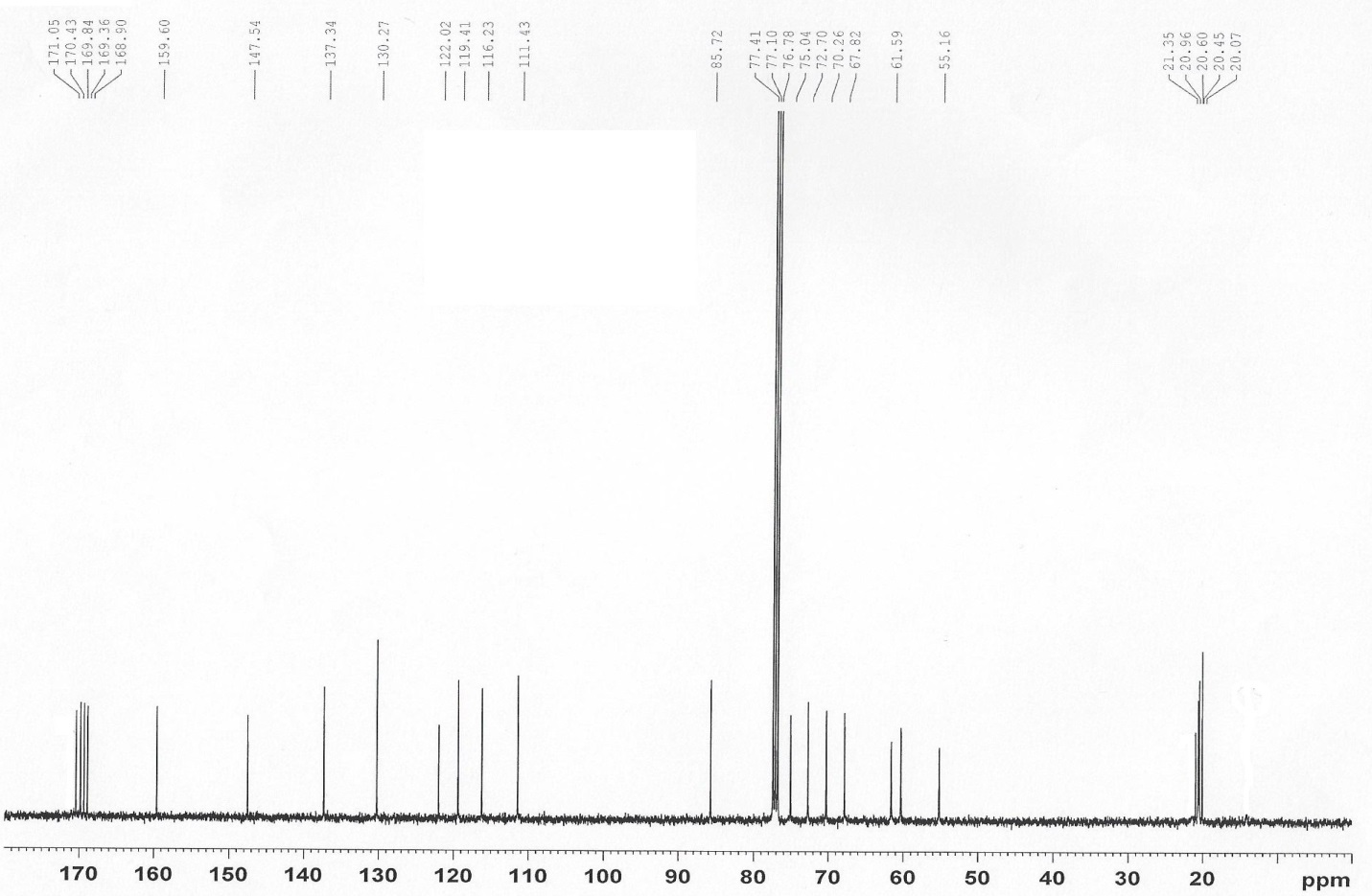


**Figure S32**. ^13^C NMR spectrum of **3dk** in CDCl_3_


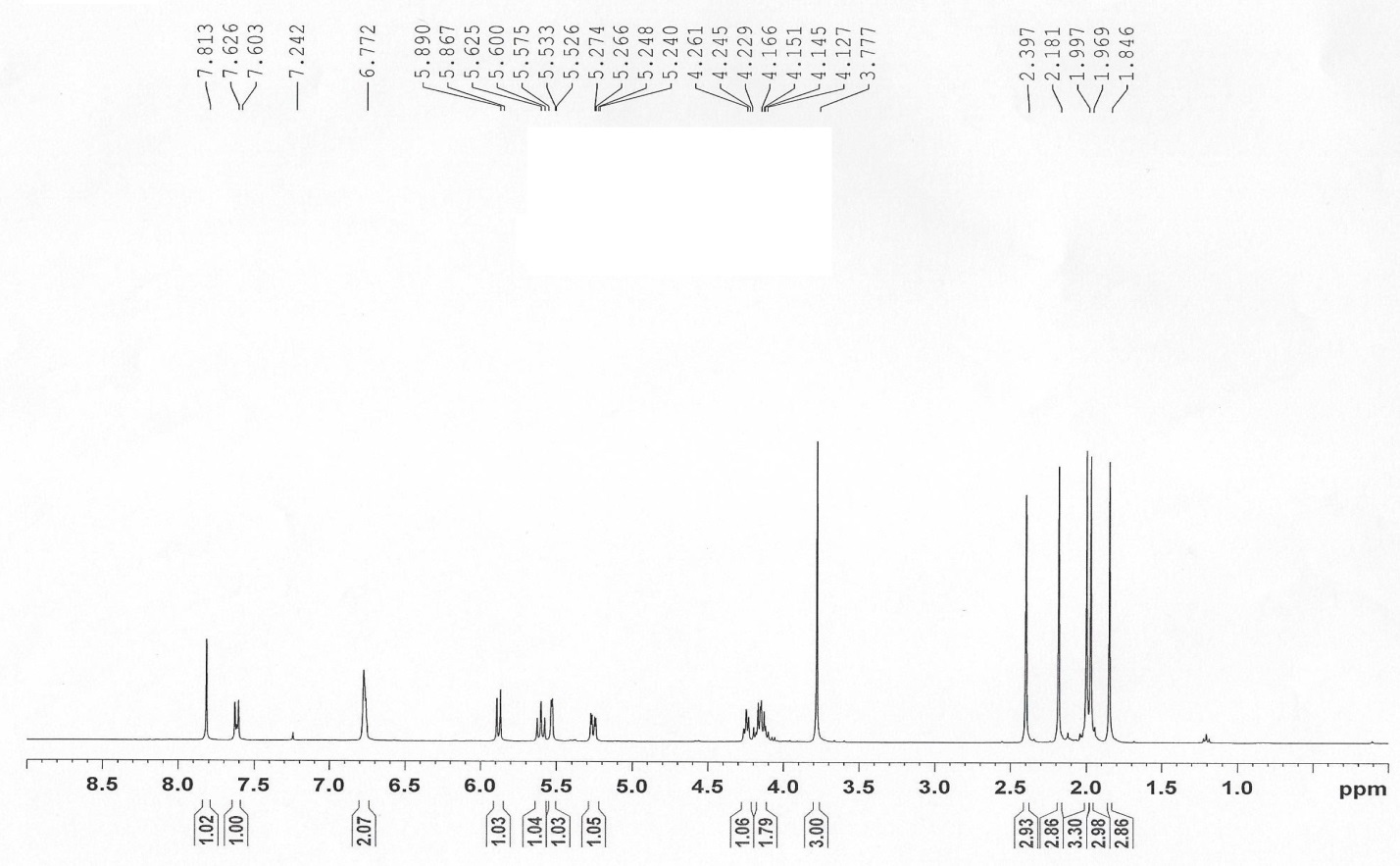


**Figure S33**. ^1^H NMR spectrum of **3ek** in CDCl_3_


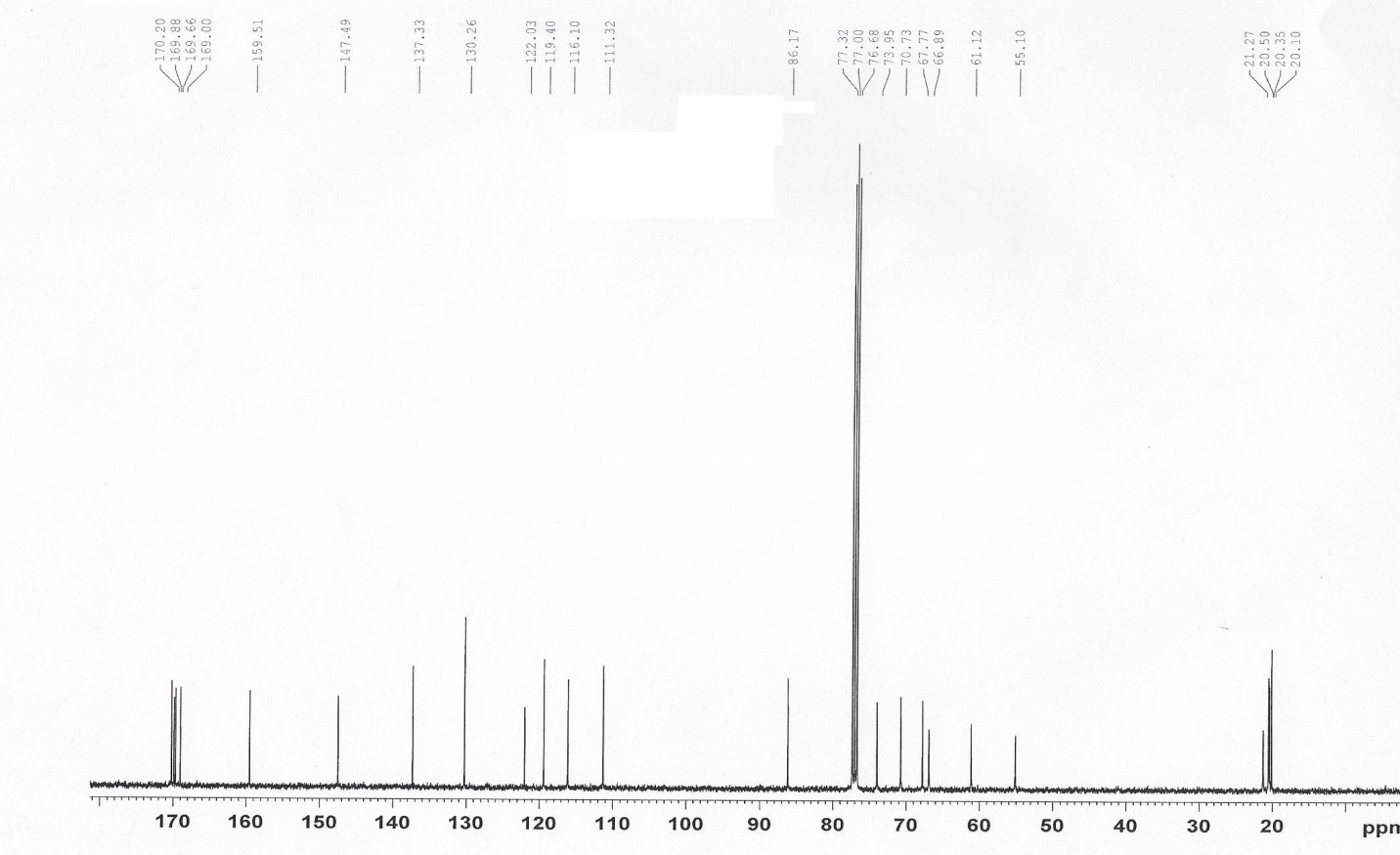


**Figure S34**. ^13^C NMR spectrum of **3ek** in CDCl_3_
